# Emerging Evidence for Pleiotropism of Eosinophils

**DOI:** 10.3390/ijms22137075

**Published:** 2021-06-30

**Authors:** José M. Rodrigo-Muñoz, Marta Gil-Martínez, Beatriz Sastre, Victoria del Pozo

**Affiliations:** 1 Immunoallergy Laboratory, Immunology Department, Instituto de Investigación Sanitaria Fundación Jiménez Díaz (IIS-FJD), Avenida Reyes Católicos, 28040 Madrid, Spain; jose.rodrigom@quironsalud.es (J.M.R.-M.); marta.gilm@quironsalud.es (M.G.-M.); 2 CIBER de Enfermedades Respiratorias (CIBERES), Av. de Monforte de Lemos, 28029 Madrid, Spain; 3 Medicine Department, Universidad Autónoma de Madrid, 28029 Madrid, Spain

**Keywords:** eosinophil, sub-phenotypes, eosinophil extracellular traps, biologic treatment

## Abstract

Eosinophils are complex granulocytes with the capacity to react upon diverse stimuli due to their numerous and variable surface receptors, which allows them to respond in very different manners. Traditionally believed to be only part of parasitic and allergic/asthmatic immune responses, as scientific studies arise, the paradigm about these cells is continuously changing, adding layers of complexity to their roles in homeostasis and disease. Developing principally in the bone marrow by the action of IL-5 and granulocyte macrophage colony-stimulating factor GM-CSF, eosinophils migrate from the blood to very different organs, performing multiple functions in tissue homeostasis as in the gastrointestinal tract, thymus, uterus, mammary glands, liver, and skeletal muscle. In organs such as the lungs and gastrointestinal tract, eosinophils are able to act as immune regulatory cells and also to perform direct actions against parasites, and bacteria, where novel mechanisms of immune defense as extracellular DNA traps are key factors. Besides, eosinophils, are of importance in an effective response against viral pathogens by their nuclease enzymatic activity and have been lately described as involved in severe acute respiratory syndrome coronavirus SARS-CoV-2 immunity. The pleiotropic role of eosinophils is sustained because eosinophils can be also detrimental to human physiology, for example, in diseases like allergies, asthma, and eosinophilic esophagitis, where exosomes can be significant pathophysiologic units. These eosinophilic pathologies, require specific treatments by eosinophils control, such as new monoclonal antibodies like mepolizumab, reslizumab, and benralizumab. In this review, we describe the roles of eosinophils as effectors and regulatory cells and their involvement in pathological disorders and treatment.

## 1. Introduction

The term eosinophil was coined in 1879 by Paul Ehrlich to describe cells in the blood that could be easily identified when stained by the dye eosin. Although eosinophils are largely evolutionarily conserved, suggesting important physiological functions, their precise role is not well understood. Eosinophils have been traditionally recognized as cytotoxic effector cells though recent studies have challenged this simplistic view of eosinophils and their function and have led to a better understanding of the role of these cells as immunomodulators and actors in the metabolic homeostasis.

Similarly, recent developments have allowed a more nuanced view of how eosinophils contribute to the pathogenesis of different diseases, including allergic rhinitis, asthma eosinophilic chronic rhinosinusitis, esophagitis, atopic dermatitis, myopathies, and hypereosinophilic syndrome.

All this makes eosinophils an attractive target for therapeutic interventions in an expanding number of clinical areas. Given the availability of new eosinophil-targeted depletion therapies, there is renewed interest in understanding eosinophil function. Hence, profound knowledge of these cells is required. In this review, we explore multiple classical and novel biological aspects of eosinophils.

## 2. Unraveling the Complex Biology of Eosinophils: From Development to Traffic

Eosinophils were probably observed for the first time by Wharton-Jones in 1846 [1], although as previously said, it was not until later in 1879 when eosin dye was established for the identification of these cells [2]. Eosinophils can be easily differentiated from other cells, such as neutrophils and basophils, based on their morphology and brightly brick-red appearance when stained with hematoxylin and eosin [3].

**Figure 1:**
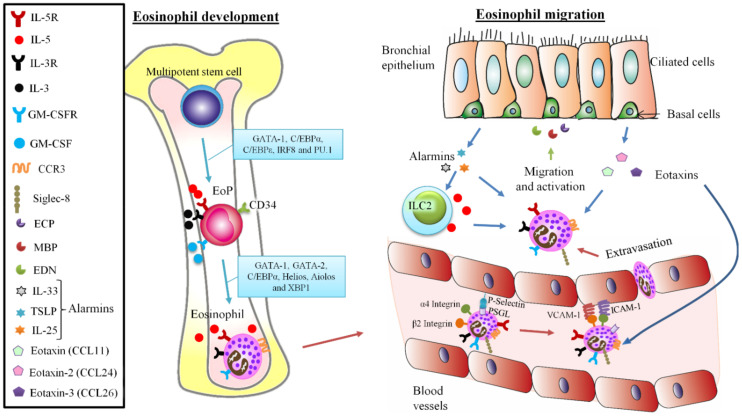
Eosinophils development and migration. Eosinophils develop mainly in the bone marrow from stem cells turning into eosinophil progenitors (EoP) expressing CD34 by the action of transcription factors (GATA-1, C/EBPα, C/EBPε, IRF8 and PU.1). By IL-5, IL-3 and granulocyte macrophage colony-stimulating factor (GM-CSF) interaction over their specific receptors (IL-5R, IL-3R and GM-CSFR) eosinophils turn into final state through the effect of the transcription factors GATA-1, GATA-2, C/EBPα, Helios, Aiolos and XBP1, and flow into the blood system, being maintained by IL-5 and GM-CSF. From blood, eosinophils transmigrate the vessels, first by performing adhesion by interaction of P-Selectin Ligand (PSGL) with the vascular P-Selectin, and secondly, interacting with the blood vessel molecules vascular cell adhesion molecule 1 (VCAM-1) and intercellular adhesion molecule 1 (ICAM-1) using their integrins (α4 and β2 respectively), allowing the rolling and extravasation of eosinophils attracted to the lung epithelium due to the combined action of eotaxin-1 (CCL11), eotaxin-2 (CCL24) and eotaxin-3 (CCL26) towards the eosinophilic CC-chemokine receptors-3 (CCR3) receptor. The migration is also sustained by IL-5 secretion produced in type 2 innate lymphoid cells (ILC2s). Both ILC2s and eosinophils themselves are activated by epithelial alarmins as IL-33, thymic stromal lymphopoietin (TSLP) and IL-25, inducing eosinophil degranulation of eosinophil cationic protein (ECP), major basic proteins (MBP) and eosinophil-derived neurotoxin (EDN), which produce epithelial remodeling.

**Figure 2:**
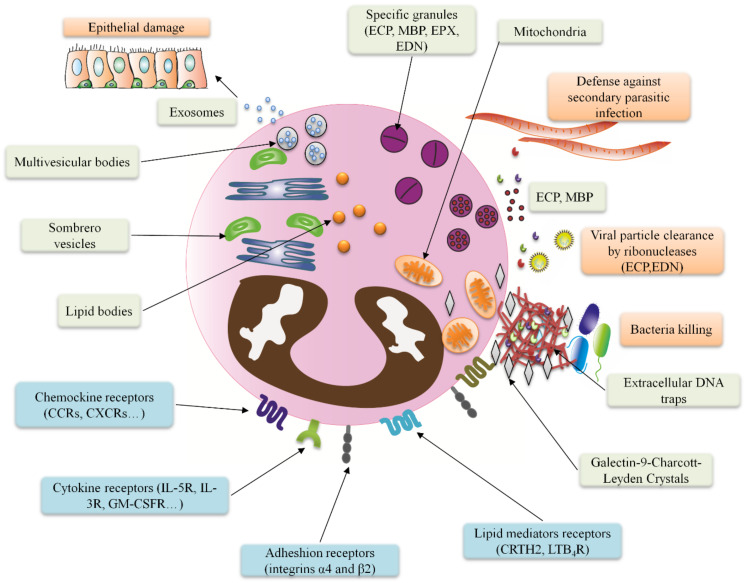
Eosinophil granules and mechanisms of action. Eosinophils have on their surface diverse receptors including chemokine receptors (CCRs, CXCRs…), lipid mediators’ receptors (CRTH2, LTB4R) or cytokine receptors (IL5Rα, IL-3R and GM-CSFR). These receptors alongside adhesion molecules as integrins α4 and β2 allow eosinophils to migrate and react against very variable stimulus. Eosinophil responses are performed thanks to their granule content. First, enzymatic content as ECP, MBP and EDN are secreted from the specific granules by piecemeal degranulation (mediated by sombrero vesicle transport) allowing viral clearance by their ribonuclease activity and the reaction against secondary parasitic infections. Besides, eosinophils kill bacteria by release of their DNA content, from the nucleus or the mitochondria, forming extracellular DNA traps with bound enzymes. Alongside the DNA traps, sometimes Charcot-Leyden Crystals are released. Finally, eosinophils secrete exosomes from multivesicular bodies fusing to the cell membrane, which are involved in epithelial damage.

Commonly, eosinophils are a kind of white blood cells, measuring 10–20 µm in diameter, with a bilobed nucleus [4] (Figure 1 and Figure 2). In homeostatic situations, they circulate in the bloodstream ranging from 0 to 500 eosinophils per microliter [5]. These cells have a very active metabolism, and they are characterized by numerous intracellular secretory granules contained in the cytoplasm, which store the majority of cationic granule proteins and a variety of cytokines, chemokines, and growth factors, and from which they are mobilized and released in response to cell activation [6].

The most peculiar characteristic of eosinophils is in the presence of specific granules, also known as secondary granules, which consist of a dense crystalline core, a unique feature of eosinophils, embedded in a matrix and surrounded by a membrane [7]. These granules contain a large number of mediators that are capable of inducing inflammation and/or tissue damage, predominantly cationic (basic) proteins. Major basic proteins are located in the core: MBP-1, encoded by *PRG2*; and MBP2, a homolog of MBP-1 which has been described as being less cationic and for being strictly eosinophilic specific (as MBP-1 is also present in basophils and mast cells) [8], being both proteins involved in asthma pathogenesis; while the matrix contains eosinophil cationic protein (ECP, encoded by *RNASE3*), eosinophil peroxidase (EPX, encoded by *EPX*), and eosinophil-derived neurotoxin (EDN, encoded by *RNASE2*) with roles in airway remodeling in asthma [9] (Figure 1 and Figure 2). Moreover, it is well documented that proteolytic processing of granule cationic proteins during eosinophil cell maturation, from bone marrow progenitors, is required both for specific granule formation [10]. Indeed, granule formation is a fundamental event in eosinophil development as shown in a report where the combined loss of MBP and EPX causes disruption of eosinophilopoiesis; and another study showing that proteolytic processing of granule proteins is required for eosinophil survival and granule formation [11].

Besides their specific granules, Charcot-Leyden crystal protein (a member of the carbohydrate-binding family of galectin-10) is another type of eosinophil content, characterized for forming extracellular hexagonal bipyramidal crystals, which exhibit lysophospholipase activity and have been identified as a hallmark of eosinophil involvement in allergic inflammation [12]. Finally, other types of secreted builds have been identified in eosinophils, such as lipid bodies are particularly important due to their involvement in the production of eicosanoids, including cysteinyl leukotrienes, prostaglandins, and thromboxane [13]; or exosomes, extracellular vesicles that have been described as involved in asthma pathogenesis [14] (Figure 2).

Prior to circulation and recruitment to the tissue eosinophils, like all other myeloid blood cell lineages, are produced and developed in the bone marrow from multipotent hematopoietic stem cells, which creates a population of eosinophil lineage–committed progenitor cells, termed EoPs, which are capable of terminally differentiating into mature eosinophils (Eos) (Figure 1) [15]. In humans, EoPs are identified by surface expression of CD34, CD38, and interleukin (IL) 5 (IL-5) receptor alpha (IL-5Rα, or CD125) [16]. Eosinophils can also develop from CD34^+^ progenitor cells found outside the bone marrow, blood, and notably in lung tissue [17,18]. Likewise, increased numbers of CD34^+^/IL-5Rα^+^ eosinophil precursors have also been identified in the bronchial mucosa of asthmatics compared to non-asthmatic control individuals [19] (Figure 1 and Figure 2).

Eosinophil lineage commitment is regulated by the ordered interaction of multiple transcription factors, including GATA factors (GATA-1 and GATA-2), ETS-family member PU.1, the CCAAT-enhancing binding protein (C/EBP) family members C/EBPα and C/EBPε, the two nuclear factors friend of GATA-1 (FOG-1; Zfpm1) and interferon regulatory factor 8 (IRF8; Irf8 or Icsbp), X-box binding protein 1 (XBP1), and members of the Ikaros zinc finger (IkZF) family, Helios (Ikzf2) and Aiolos (Ikzf3) [20,21]. GATA-1 is essential and plays a crucial role in eosinophil differentiation, as disruption of the GATA-1 gene in mice results in a strain completely devoid of eosinophils. Recent studies highlighted the importance of this transcription factor, key in defining a population of progenitors named erythroid/megakaryocyte-primed multipotent progenitors (EMPP) which are able to generate megakaryocytes, erythroid cells, basophils/mast cells, and eosinophils, are CD131^+^ cells and deriving from CD38–135^-^ progenitors [22,23,24].

In addition to regulation by transcription factors, cytokines support the development of the eosinophil lineage, and indeed, the action of IL-5, IL-3, and granulocyte-macrophage colony-stimulating factor (GM-CSF) is vital in eosinophils development—[25,26]. IL-5 is the most specific cytokine for eosinophils and acts at multiple functional levels and time points during their lifespan [27]. In addition to promoting proliferation, differentiation, and maturation of IL-5Rα-expressing eosinophil precursors in the bone marrow, IL-5 contributes to the release of eosinophils into the bloodstream and the activation and survival of mature eosinophils on the periphery [28]. Although the most common source of IL-5 is Type 2 helper CD4^+^ T (Th2) cells, mast cells and group 2 innate lymphoid cells (ILC2s) are able to release IL-5 [29]. IL-5 acts synergistically with eotaxins, a variety of C-C motif chemokine ligands (CCLs), which are selective chemotactic factors that mediate the migration and recruitment of these cells into the body tissues and their activation [30]. Eotaxin (CCL11), eotaxin-2 (CCL24), and eotaxin-3 (CCL26), among others, bind to CC-chemokine receptors-3 (CCR3) on eosinophil cell membranes and can induce chemotaxis in allergic inflammation [31] (Figure 1). 5-oxo 6, 8, 11, 14-eicosatetraenoic acid (5-oxo-ETE) is another eosinophil chemoattractant [32].

Although less specific than IL-5 for eosinophils, the cytokines IL-3 and (GM-CSF) are also implicated in the activation and survival of tissue eosinophils through induction of Bcl-xL expression [33,34]. Moreover, epithelial cell-derived alarmins, IL-25 (also known as IL-17E), IL-33, and (TSLP) promote eosinophilopoiesis by increasing IL-5 production by ILC2 cells [35]. Interestingly, the action of these alarmins over eosinophilopoiesis is not only indirect due to IL-5 secretion by ILC2s, as IL-33 has been described to precede and promote the signaling by IL-5 in the process of eosinophil development [36]. Finally, it is worth noting the role of molecules such as microRNAs in the regulation of eosinophil thymic stromal lymphopoietin ontogeny [37].

Like all leukocytes, the eosinophil displays a wide range of surface molecules and receptors, which enables them to integrate with the innate and adaptive immune systems [38]. The heterodimeric receptor for interleukin 5 (IL-5) is thought to be the most important cytokine receptor expressed by eosinophils because IL-5 has a central and predominant role in all aspects of eosinophil development, activation, and survival [30,39]. Other cell surface structures are relatively specific for eosinophils, such as CC-chemokine receptor 3 (CCR3), which mediates eosinophil chemotaxis in response to eotaxins, and sialic acid-binding immunoglobulin-like lectin 8 (Siglec-8), whose engagement induces apoptosis to activated eosinophils [40,41] (Figure 1). Epidermal growth factor (EGF) module containing mucin-like hormone-like receptor 1 (EMR1) is a surface receptor that is completely unique to the eosinophil [42]. The vast array of receptors present in these granulocytes makes them very versatile cells, with a capacity to react to stimulus, co-stimulate cells in antigen presentation, and migrate to the tissues at physiological and pathological states [43]. Besides, this kind of cell is also equipped with certain intracellular receptors that regulate function (e.g., some toll-like receptors and the glucocorticoid receptor) likewise with a wide number of surface receptors [43,44,45].

Eosinophils are released into the peripheral blood (they have an approximate half-life of 8 to 18 h) in a phenotypically mature state before migrating to tissues where they can persist for at least several weeks under homeostatic conditions [46]. In healthy individuals, most eosinophils are found throughout the gastrointestinal (GI) tract, with the notable exception of the esophagus, and in other locations such as the mammary gland, uterus, thymus, bone marrow, and adipose tissue [47]. Extravasation out of circulation and into tissue sites is dependent on the function of integrins and their counter-ligands on activated endothelium and eosinophil-selective chemoattractants such as the eotaxins, mentioned above [5]. In the same way, under inflammatory conditions such as allergic inflammation and asthma, an array of chemotactic proteins participate in eosinophil recruitment to the site of inflammation [48]. Notably, the eotaxin chemokines are markedly induced by IL-13, providing a synergistic mechanism by which Th2 cells and ILC2s, coproducing IL-5 and IL-13, regulate tissue eosinophilia [49].

## 3. The Eosinophil Immune Response Mechanisms against Diverse Pathogens

Eosinophils are highly versatile immune cells that are capable of acting against a wide arrange of pathogens, from those as small ones as a virus [50], to bigger ones such as parasites [51] by performing mechanisms of action including cytokine synthesis, classical degranulation, and release of exosomes and eosinophil extracellular traps (EETs) tools, being classical and recently discovered methods utilized by these resourceful innate cells.

### 3.1. Revisiting Eosinophil’s Role against Parasites

Traditionally, eosinophils were believed to be only involved in parasitic infections and allergic diseases [1]. Indeed, several studies have proved that eosinophils and their granule content, combined with their ability to secrete cytokines are major effectors in host defense against parasitic infections, being able to control both in vitro as in vivo, diverse parasites including *Trichinella* or *Nippostrongylus*, mainly acting during the secondary infection by antibody-dependent cellular cytotoxicity (ADCC) and release of granule enzymes [52]. Undoubtedly, one of the major features of eosinophils is their granules and their enzymatic content [7]. The release of the eosinophil granules occurs through exocytosis, cytolysis, or via piecemeal degranulation. The first mechanism described was compound exocytosis, a widely described anti-parasitic mechanism displayed by eosinophils in vitro, which consists of the release of their full granule content by the fusion of the granule membrane with the cell membrane [53]. Conversely, piecemeal degranulation allows granule secretion selectively by a cytoplasmic membrane-vesicular tubular network, that transport the granules until they fuse to the cell membrane [54], in a process where CD63 is involved [55], with an important role in the secretion of MBP-1 against *Schistosoma mansoni* infection, proving that eosinophil are able to perform defense mechanisms against parasitic and infections, both by “classical” pathways and by more recently described processes [56] (Figure 2). It is of note that this type of degranulation is implicated in the sorting and secretion of specific cytokines in response to stimuli, as it occurs for IL-4 containing granules, which also bear IL-4R, and which are mobilized in response to eotaxin-1 [57]. The presence of specific receptors on the granule surfaces gives them the capacity to release specific molecules, as observed for the secretion of ECP upon granule stimulation with leukotriene (LT)C4, -D4, and -E4 of the granule receptors cysteinyl(cys)-LT1R, cysLT2R, and purinergic P2Y12R [58]. The importance of correct ECP secretion against pathogens lies in several studies of *RNASE3* (ECP) single nucleotide polymorphisms (SNPs), as studies have described an association between SNPs of *RNASE3* that abolish its cytotoxic activity, with the appearance of severe or cerebral malaria (caused by *Plasmodium falciparum*) in populations of Ghana and Senegal respectively [59,60].

Nevertheless, regarding eosinophil’s role in defense against parasites, novel studies have determined that the initial dogma that associated eosinophil with parasitic clearance is more complicated than expected at the beginning. The development of this knowledge was linked to the use of eosinophil ablated mouse models, showing that eosinophils depletion does not alter the immune responses against primary infection with several parasites [52]. Surprisingly, eosinophils can even promote parasitic survival, as *Trichinella spiralis* larvae die in the skeletal muscle of mice without eosinophils, correlating with higher IFN-γ and lower IL-4. Eosinophil’s promotion of larvae survival is mediated by IL-10 secretion, which activates IL-10^+^ dendritic cells and CD4^+^ IL-10^+^ T lymphocytes that inhibit inducible nitric oxide (NO) synthase, protecting parasites [61,62]. These results emphasize a very complex interplay between eosinophils and parasites, which depend on the moment and place of infection.

### 3.2. Eosinophil Responses against Bacteria, the Involvement of Extracellular Traps

Finally, the last method of eosinophil degranulation is cytolysis, which consists of cellular death involving the necroptotic pathway [63], releasing their intact granules, which can be reactive to leukotrienes [58], due to the presence of specific receptors coupled to G proteins (CPGRs), or recognizing specific cytokines (IFN-γ) [64]. The eosinophils’ cytolysis process includes the release of genetic material alongside the granules [65]; this DNA forms nets, also known as eosinophil extracellular traps (EETs). Interestingly, not all the EETs are released through cell lysis, as authors have shown that eosinophils are able to expel their mitochondrial DNA without dying, when facing bacteria or fungi, since MBP and ECP are bound to these nets, highlighting that eosinophils do not only act in parasitic infections and that they are able to act against other pathogens like bacteria and fungi [66,67,68] (Figure 2). Indeed, it has been extensively described that the common mechanisms performed against parasites such as enzyme and cytokine release are also functional against a wide array of different pathogens. This kind of eosinophilic responses have been mainly described in the gut and in the lungs, showing that in vitro, eosinophils can react against diverse kind of bacteria including *Escherichia coli*, *Clostridium perfringens* and *Streptococcus pneumoniae* [69,70] and demonstrating their antibacterial role in vivo against *Pseudomonas aeruginosa* or *Staphylococcus aureus* releasing their granule content as ECP [71,72].

Although EETs are effective against pathogens, the eosinophil DNA traps have also been associated with diseases where eosinophils are involved such as allergic asthma. In this pathology, DNA traps were detected by bronchial biopsy [73] from severe eosinophilic asthma patients, in which EETs were highly secreted compared to non-severe asthma; moreover, their levels correlated with activation of eosinophils and airway epithelial cells [74]. Another example is active eosinophilic esophagitis samples where these nets presented the association with Charcot-Leyden crystals [75]. Indeed, Charcot-Leyden crystals have been related to eosinophil extracellular trap cell death (ETosis). In a study by Ueki *et al.*, the authors described that during ETosis, cytoplasmic galectin-10 is released alongside granules and extracellular traps, contributing to the formation of Charcot-Leyden crystals extracellularly [12] (Figure 2). Recently, it has even been described that galectin-10 is mainly stored free in the cytoplasm of the eosinophils, not being stored in granules nor secreted by regular degranulation [76]. Nevertheless, controversy persists whether the eosinophil remains alive or dies after releasing the DNA trap, and it seems that the most important factor for either to happen is the nature and timing of the stimulus [66,77].

### 3.3. Eosinophilic Responses against Virus and Current Knowledge about Eosinophils Involvement in COVID-19

In the last years, eosinophils have also been described as good effectors against viral pathogens, a process that is mainly related to the presence of nucleases such as ECP or EDN as their granule content [78] (Figure 2). This antiviral activity of eosinophils has been detected against the respiratory syncytial virus (RSV) in a mechanism involving toll-like receptor (TLR)7/MyD88 signaling and ribonucleases release [79]. Nonetheless, eosinophil nucleases are not the only weapon against viruses, as these cells promote immunity against influenza A virus by inducing CD8^+^ T cell proliferation and activation [80], and face human parainfluenza virus by secreting nitric oxide through TLR7 activation [81]. Furthermore, eosinophils recruited by *Aspergillus fumigatus* protect against lethal pneumovirus infection, although these responses might depend on airway location, as exposure to *Alternaria alternata* at the nasal mucosa recruited inflammatory eosinophils with no antiviral effect against influenza infection [50,82]. More exhaustive reviews on eosinophils role against the virus can be found elsewhere [83,84] (Table 1).

Due to the current COVID-19 pandemic state, it is of interest to describe how eosinophils affect and are affected by severe acute respiratory syndrome coronavirus 2 (SARS-CoV-2). Symptomatic COVID-19 patients were characterized by blood eosinophilia which was associated with lower C-reactive protein (CRP) and better disease outcome, indicating a protective role for eosinophils in the first stages of COVID-19 [85]. Pre-existing or developed eosinophilia (up to the seventh day of infection) was associated with decreased mortality and better prognosis [86,87,88]. The protective role of eosinophils depends on race/ethnicity, as white and Hispanic patients with a high eosinophil percentage have higher odds of survival [89].

Conversely, having eosinopenia, or developing it in the first compasses of the disease is an indicator of poor prognosis, and the worst disease course, and eosinophil and neutrophil counts can be used to diagnose COVID-19 [90]. This association of eosinopenia with fever and pneumonia was confirmed in a young and middle-aged cohort [91] and in an elderly cohort, predicting intensive care unit (ICU) risk and serum cytokine increase [92]. Eosinophil/lymphocyte counts are reduced in patients with longer hospitalizations and worse outcomes [93,94], while recovery of cell levels is associated with symptoms of amelioration [95]. On the other hand, if eosinopenia is sustained and worsened by progressive eosinophil reduction, it causes higher mortality, tissue damage, and a higher presence of coagulation disorder markers [96]. Nevertheless, in severe COVID-19 eosinophil counts, eotaxin-2 and IL-5 blood levels are higher compared to moderate disease [97]. There are certain signs of type 2 activation, in ICU admitted COVID-19 patients, including eosinophil degranulation [98] (Table 1). Eosinophils involved in the acute phase of COVID-19 are lung resident CD62L^+^ able to respond to interferon (IFN)-γ, causing eosinophilic expansion preceding lung hyperinflammation, which might be related to the most severe cases [99]. These results highlight that higher eosinophil levels at the beginning of infection are good for prognosis, while a slow reduction of eosinophils after clearance is required, as these might otherwise cause tissue damage in the late phase [100].

Eosinophils and other blood cell subsets could be COVID-19 biomarkers, as higher serum cytokine levels and decreased lymphocyte counts are associated with worse COVID-19 [101]. Eosinopenia or the ratio between eosinophil and polymorphonuclear neutrophils can be used to diagnose COVID-19 on the day of SARS-CoV-2 infection suspicion when polymerase chain reaction (PCR) kits are limited [102,103,104,105]. Nevertheless, differences in eosinophil counts are very small and heterogeneous [106], and current techniques such as RT-PCR or viral antigens/SARS-CoV-2 antibodies detection are still the Gold Standard techniques in COVID-19 diagnosis [107].

With respect to eosinophilic disorders and COVID-19, the presence of T2 asthma was not a risk factor for worse COVID-19 as compared to other comorbidities as COPD [86,108]. This absence of association may be due to a reduction in epithelial angiotensin-converting enzyme 2 (ACE2, (the receptor used by SARS-CoV-2 for infection) in atopic dermatitis and allergic asthmatic children and adults [109]. Indeed, asthmatics with higher epithelial ACE2 expression were characterized by lower blood eosinophil counts and higher expression of IFN-related genes [110] (Table 1).

Nowadays, biological treatments in asthma including omalizumab, mepolizumab, benralizumab, dupilumab, and reslizumab target against T2 specific molecules. Asthmatics treated with biological drugs did not present a higher risk of SARS-CoV-2 infection or worse COVID-19 [111,112,113], only being one study showing poor disease course but the authors were unclear if it is due to biological treatment, comorbidities, severe asthma, or a combination [114]. Even benralizumab (causes eosinopenia targeting IL-5R) case reports have shown no disease worsening [115,116,117].

**Table 1:** Eosinophils and COVID-19.

Main Findings	Number of Subjects	References
Blood eosinophilia is associated with good COVID-19 prognosis	314	[85]
	951	[86]
	9644	[87]
	10	[88]
	95	[95]
	4252	[89]
Higher eosinophil counts in severe COVID-19	135	[97]
	15	[98]
	37	[99]
T2 diseases are not associated to COVID-19	189	[108]
Blood eosinopenia is a marker of worst COVID-19 disease course	324	[90]
	95	[101]
	37	[91]
	96	[93]
	121	[105]
	40	[94]
	190	[96]
	294	[118]
	94	[92]
	429	[102]
	174	[103]
	37	[104]
ACE2 receptor is reduced in asthma	365	[109]
	66	[110]
Biological asthma treatment does not affect COVID-19	676	[111]
	545	[112]
	1504	[113]
	2 cases	[115]
	2 cases	[116]
	1 case	[117]
Biological asthma treatment might have worst COVID-19 outcome	634	[114]
ACE2 = Angiotensin-converting enzyme 2. COVID-19 = Coronavirus disease 2019.		

First vaccines created against the SARS-CoV pandemic (2002) were characterized by eosinophil accumulation and T2 activation in the lung, caused by SARS-CoV-1 virus nucleocapsid protein of coadjuvant alum [119,120]. This could be prevented by the addition of delta inulin adjuvants or TLR agonists, skewing immune responses toward T1, inducing immunization without recruiting eosinophils [121,122]. Although past vaccines induced eosinophil disease, current SARS-CoV-2 vaccines have not caused lung eosinophil infiltration and accumulation to date [123].

### 3.4. Exosomes from Eosinophils Contribute to Asthma Hallmarks

The role of eosinophils in the orchestration of immune responses was identified in past years. During immune responses, the transmission of signals and molecules between cells is the key to the correct management of the host defenses. Regarding these events, the release of exosomes, a mechanism of eosinophil communication, has been described in recent years. Exosomes are extracellular vesicles (EVs) formed by budding of the cell membrane encapsulating proteins and nucleic acid, such as microRNAs (miRNAs). Exosomes are characterized by the presence of surface receptors, which allow them to fuse to receptor cells and modify their functions [124]. In 2015, we described for the first time that eosinophils can secrete functional exosomes in response to IFN-γ. These exosomes contain eosinophilic proteins such as ECP, eosinophil peroxidase (EPO), and MBP, and the secretion of exosomes is higher in eosinophils from asthmatics compared to healthy individuals [14]. These results were later confirmed, revealing that eosinophils secrete EVs expressing CD63 and CD9 in response to other stimuli such as CCL11 (eotaxin-1) and tumor necrosis factor alpha (TNF-α) [125]. Functionally, exosomes from asthmatic eosinophils autocrinally activate eosinophils, augmenting eosinophils adhesion and migration capacities through upregulation of intercellular adhesion molecule 1 (ICAM1) and integrin α2, and increasing their release of reactive oxygen species (ROS) and NO [126]. Moreover, eosinophil-derived exosomes, participate in asthmatic airway remodeling (Figure 2), as it was shown that when derived from asthmatics’ eosinophils, exosomes cause small airway epithelial cell injury through apoptosis, increasing remodeling and inflammation-related gene expression (*POSTN*, *CCL26,* and *TNF*), while also elevating bronchial smooth muscle cell proliferation mediated by pERK, and augmenting expression of *CCR3* and *VEGF* in these cells [127]. In addition to exosome function in asthma pathophysiology, the miRNA content in eosinophils can be used as a biomarker for asthma diagnosis, expanding their role in this disease [128,129]. Although these events have only been described in asthma, it is possible that eosinophils secrete exosomes also during immune responses against diverse pathogens, but until now, no data has been published regarding this matter, which opens a new promising field for scientific research and discovery.

## 4. Role of Eosinophils as Effector Cells in Homeostasis

**Figure 3:**
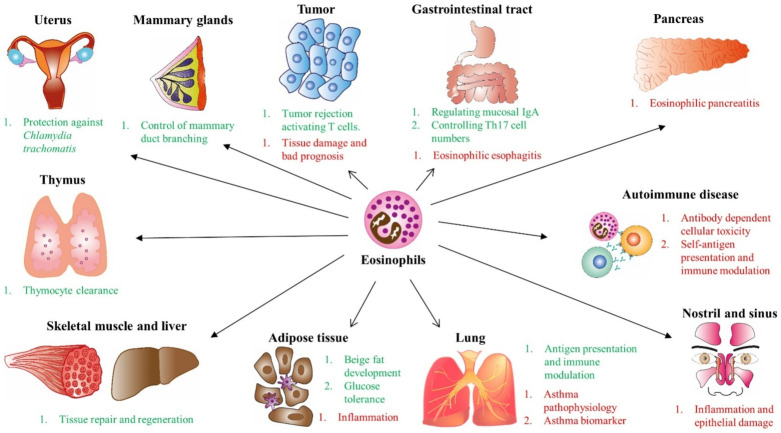
Homeostatic and pathophysiological functions of eosinophils. Eosinophils’ versatility makes these cells be able to both regulate homeostasis of diverse organs including, control of mammary duct branching, induction of protection against *Chlamydia trachomatis* in the uterus, clearance of apoptotic thymocytes in the thymus and tissue repair and regeneration of both skeletal muscle and liver by IL-4. In other organs, eosinophils have been described to perform detrimental roles, such as inducing eosinophilic pancreatitis, being involved in antibody dependent cellular toxicity and self-antigen presentation and immune modulation in the context of autoimmune diseases, while also causing inflammation and epithelial damage in chronic rhinosinusitis. In some organs, eosinophils perform both homeostatic as prejudicial roles, as in the lungs where eosinophils perform pathogen antigen presentation and immune modulation but being also related to asthma pathophysiology. In the adipose tissue, eosinophils participate in beige fat development and glucose tolerance, while inducing also inflammation. In the gastrointestinal tract they regulate mucosal IgA and Th17 cell numbers, inducing eosinophilic esophagitis when over cumulated, and finally, in tumors they both activate tumor rejection by T cells, and induces tissue damage.

The role of eosinophils in health can be studied, either by mouse knockout models or in patients receiving an eosinophil-depleting biologic drug [130]. Eosinophils are found in several tissues and organs and are maintained by type 2 innate lymphoid cells (ILC2s) through IL-5 in response to circadian rhythms and intake [131]. The gastrointestinal tract and the esophagus have a high eosinophil presence, regulating mucosal IgA secretion in mice by plasma cells through IL-1β production [132] (Figure 3). Eosinophils are required to control Th17 cell numbers by IL-1R antagonist expression and in Treg differentiation [133] by transforming growth factor (TGF)-β1 and retinoic acid levels [134,135]. Nonetheless, when eosinophils are accumulated in the esophagus, they can cause eosinophilic esophagitis, where eosinophils are recruited in response to IL-13, CCL26/eotaxin-3, and TGF-β, causing inflammation [136].

Eosinophils have been involved in mammary duct branching, both in puberty and in pregnancy, releasing signaling molecules such as CCL6, amphiregulin, and TGF-β [137,138]. Interestingly, these cells are recruited into the uterus during the estrus cycle and are important in preventing *Chlamydia trachomatis* infections by increasing endometrial stromal cell proliferation through IL-4 [139]. Metabolism of mouse models are regulated by eosinophils, being these cells required in glucose tolerance, beige fat development, and inflammation in adipose tissue through M2 macrophage polarization by IL-4 secretion [140,141,142,143]. Metabolic cold-induced processes also involve eosinophil recruitment to adipose tissue by CCL11 for thermoregulation [144].

Eosinophils’ role in immune regulation starts at the thymus, where they control the clearance of apoptotic thymocytes [145] (Figure 3). In the lungs, eosinophils act as antigen-presenting cells, expressing major histocompatibility complex (MHC) class II and the costimulatory proteins CD40, CD80, and CD86, activating Th2 responses [146]. In addition, eosinophils induce class switching of B cells into IgA and affect T cell recruitment to the lungs [144,147]. Indeed, eosinophils are highly versatile, producing T2 cytokines such as IL-4, IL-13 [49], T1 cytokines including IFN-γ and IL-8 [148,149], and such immunoregulatory cytokines as IL-10, TGF-β, and GM-CSF [150,151].

Finally, eosinophils participate in skeletal muscle repair, releasing IL4 and IL-13, which are required for activation of fibrocyte–adipocyte progenitors (FAPs) in myogenic differentiation [152]. IL-4 secretion induces liver regeneration, activating the proliferation of hepatocytes expressing IL-4Rα [153]. Eosinophils also regulate inflammation and repair in colon injury, upregulating s100a8 and s100a9 gene expression [154], and regulating angiogenesis by osteopontin [155]. Nevertheless, overactivated repair by eosinophils may cause an increase in tissue remodeling as in eosinophilic esophagitis and asthma [156].

All these reports demonstrate that eosinophils are very versatile cells, with roles both as innate immune cells, perpetrating defense against diverse pathogens, and as regulators of the correct physiology of several organs, being able to very different processes such as tissue repair and metabolism. It is worth noting that the majority of the homeostatic roles of eosinophils have been demonstrated in mice models, and translation into humans is pending validation.

## 5. Heterogeneity and Phenotypes of Eosinophils

Eosinophils are found in several organs, where they exert several actions in normal tissue processes and are also recruited to other areas during morphogenesis and repair. Recent studies have demonstrated the existence of eosinophil subsets and plasticity in different tissue contexts [157], supporting the hypothesis that points to the importance of the microenvironment in modulating the activity of eosinophils is defended.

Emerging data have revealed functional and phenotypic heterogeneity, classifying them in two main subtypes of eosinophils based on their maturation stage, organ location, and the morphogenetic activity of tissues [15,158]. The identification of heterogeneous phenotypes in homeostasis at baseline and within the context of eosinophilic diseases is an important current focus in eosinophil biology.

The local environment is capable of inducing changes in eosinophil phenotype depending on specific functions of the tissue. Moreover, differences in surface markers and function between eosinophils in a steady-state and under inflammatory conditions have been described [43]. Following the classification of Abdala-Valencia et al. [157], eosinophils are divided into four groups: immature eosinophils as precursors (EoP); tissue-resident eosinophils in quiescent tissues (steady-state eosinophils); eosinophils situated in an interstitial location in innate defense, acute inflammatory and transient morphogenetic contexts (Type 1); and finally, eosinophils found in epithelial contexts associated with an atypical type 2 immune response (Type 2). So, this division could be used to form two major groups: homeostatic eosinophils (hEos) and inflammatory eosinophils (iEos). Indeed, it has been observed that there are differences in the recruitment process of eosinophils to different tissues in relation to the degree of dependence on IL-5 [159]; this recruitment may be independent, partly dependent, or totally dependent on local IL-5 production in the lungs, gastrointestinal tract, or adipose tissue, respectively.

Homeostatic eosinophils usually express CCR3, Siglec-F, and CD125 [160]. In some locations, eosinophils may express CD11b, F4/80, CD69, and CD44 [132,134,140,161]. Moreover, most eosinophils of tissues express CD11c [132].

Specifically, eosinophils from the lung are of a special tissue phenotype because they share features with peripheral blood eosinophils [162]. In humans, lung-resident eosinophils express Siglec^−^8^+^CD62L^hi^IL-3R^low^ in humans [163]. In contrast, recruited inflammatory eosinophils express Siglec^−^8^+^CD62L^low^IL-3R^hi^, suggesting that homeostatic and inflammatory eosinophils probably exert different functions and effects. Moreover, unlike classical actions performed by eosinophils in an inflammatory lung disease such as asthma, lung-resident homeostatic eosinophils express several genes related to the maintenance of immune homeostasis in the lung [164], suggesting an ability of these homeostatic lung-resident eosinophils to regulate the type 2 immune response in this organ.

This heterogeneity in phenotypes and functions depending on maturation, location, and microenvironment highlights the difficulty in understanding the mechanistic process and pathologies in which eosinophils are implicated and the related complexity of therapeutic approaches.

## 6. Eosinophil Immune Dysfunction (EID)

In previous sections we have demonstrated the multifaceted functions that eosinophils develop in multiple scenarios, performing an important role in host protection against fungi, bacteria, and viruses, through multiple mechanisms and properties [84]. Moreover, their capabilities as effector cells and as antigen-presenting cells allow them to participate in multiple situations and promote several facets of homeostasis mechanisms. Likewise, tissue eosinophils exert functions in steady-state development [157,159], in the regeneration of different tissues [153,165], and in metabolic and immune homeostasis [134,140,166].

However, although multiple functions in homeostasis, protection against several pathogens, and regulation are performed by eosinophils, this cell population is associated with a myriad of inflammatory diseases [6] characterized by a relevant implication of eosinophils in its idiosyncrasy, recently named eosinophil immune dysfunctions (EID), which comprise diseases of several systems and organs with different symptoms and origin.

Before analyzing the role of eosinophils in diseases with different etiology, it is important to clarify the concept of eosinophilia. Usually, the degree of eosinophilia is defined based on the absolute eosinophil count in peripheral blood. Thus, eosinophilia is defined as greater than 500 eosinophils/mm^3^ and further categorized as mild (500–1500 cells/mm^3^), moderate (1500–5000 cells/mm^3^), or severe (>5000 cells/mm^3^) [167]. In relation to tissue eosinophilia, thresholds to consider a pathologic increase are not well defined. These thresholds differ from those of diseases involving the esophagus or stomach, and further studies are needed to determine which threshold defines disease in each area of the gastrointestinal tract [168]. In the case of esophageal eosinophilia, an eosinophil counts greater than or equal to 15 eosinophils per high-power field is the minimum for eosinophilic esophagitis diagnosis in addition to other features [169]. However, eosinophilia is defined as having 30 eosinophils per high-power field (eos/hpf) in at least 5 high-power fields in the stomach [170]. In addition, the clinical context must be taken into account.

### 6.1. Eosinophilic Gastrointestinal Diseases (EGID) and Pancreatic Disorders

Eosinophilic gastrointestinal diseases (EGID) refer to any pathology involving accumulation of an abnormal number of eosinophils in a specific region of the gastrointestinal tract, encompassing several entities such as eosinophilic esophagitis (EoE), eosinophilic gastritis, and eosinophilic colitis.

Since it was first described, the prevalence of EoE has increased in both adults and children [171]. EoE is a chronic immune-mediated esophageal disease characterized clinically by symptoms of esophageal dysfunction and histologically by eosinophil-predominant inflammation restricted to the esophagus, with biopsies of this mucosa showing 15 or more eos/hpf [169]. This disease has a complex etiology due to both genetic and environmental factors and has a strong heritability, high sibling recurrence risk, and early-life environmental exposure [172]. Although eosinophils are thought to be one of the key effector cells implicated in this and other EGIDs, eosinophils are not the selectively targeted cell by treatments, as a restrictive diet and the use of proton pump inhibitors are the most common therapeutic approach; however, reduction of eosinophil number is accompanied by an improvement in symptoms and endoscopic findings, reduction of histological disease severity and type 2 inflammation. More studies need to be performed to clarify the exact role of eosinophil in EGID, although new knowledge about microRNAs (miRNAs) linked to the evolution of this disease has been published elsewhere [173].

In the case of pancreatic disease, chronic pancreatitis (CP) is an inflammatory disorder caused by alcohol consumption, blockage of the pancreatic duct, and trypsinogen mutation [174]. In the initiation and progression of this disease, granulocyte infiltration is essential to pancreatic inflammation [175]. Neutrophils are the main cell type implicated in this disorder; however, several cases have been reported defining the presence of eosinophils in patients with pancreatitis, naming this entity as Eosinophilic Pancreatitis [176,177] (Figure 3), being a rarely occurring disorder.

### 6.2. Eosinophilia in Myeloid Neoplasms and Solid Tumors

Eosinophils’ role in human tumor biology is an active field of research [178]. The presence of eosinophils has different implications depending on the type of tumor process. Multiple studies have demonstrated that eosinophils have contradictory effects. Their number and location determined by histology correlate with beneficial effects like in breast or colon cancer and melanoma; detrimental effects such as in the case of Hodgkin lymphoma; or unknown significance like in pancreatic cancer [130]. So, in some cases, eosinophil degranulation infiltrating the tumor is crucial to tumor rejection and activation of CD8 T cells and consequent tumor killing [179,180]; alternatively, eosinophils may participate directly in tumor killing after activation by IL-33 [181]. Contrary to these results, in acute lymphoblastic leukemia, eosinophils are associated with organ and tissue damage and bad prognosis [182,183]. All these data on the presence of eosinophils in human cancer raise the possibility that the eosinophils are an important effector cell in malignancies (Figure 3).

### 6.3. Eosinophilia in Autoimmune Diseases

Due to their versatility, eosinophils could perform an important role as effector cells in many autoimmune diseases [184]. Their cytotoxic granules could contribute to tissue damage, and their capacity for antibody-dependent cellular cytotoxicity (ADCC) against mammalian targets enables them to kill host cells bound by antibodies [185] (Figure 3). Activated eosinophils are frequently found in areas of fibrogenesis, evidencing a potential profibrotic role that may add to tissue dysfunction in autoimmune diseases [186]. Moreover, eosinophils can modulate the immune response through activation of other elements of the immune response; so, eosinophils may contribute to the initiation of autoimmune responses by presenting antigens to T cells, activating them [187]. The variety and heterogeneity of their granules containing numerous cytokines may affect T cell differentiation. Otherwise, eosinophils could contribute to liver and muscle regeneration [152,153]. So, the effect of eosinophils likely depends on context and disease.

There are multiple autoimmune diseases in which a potential role of eosinophils has been demonstrated such as bullous pemphigoid, eosinophilic granulomatosis with polyangiitis (Churg-Strauss syndrome), Crohn’s disease, and primary biliary cirrhosis [188,189,190,191].

### 6.4. Eosinophilia in Lung Diseases: Atopic Diseases, Asthma and Interstitial Lung Disease

Atopic diseases comprise several pathologies with an allergic etiology, such as atopic dermatitis, chronic rhinosinusitis, and asthma.

Atopic dermatitis is the most common form of eosinophilic skin disease. In the early stages, it is characterized by atopic eczema and a T2 immune response that leads to itchiness and skin damage. However, in severe stages of this disease, the impact of treatment on eosinophilia has not shown a clinical impact [192], suggesting that the role of other immune cells such as lymphocytes and mast cells is more crucial to the progression of this disease.

Chronic rhinosinusitis presents two phenotypes based on the absence (CRSsNP) or presence (CRSwNP) of nasal polyps [193]. Most patients in the former group are pathophysiologically characterized by accumulation and prolonged survival of eosinophils in sino-nasal mucosa [194], releasing pro-inflammatory mediators that could be partially responsible for the clinical consequences of chronic inflammation (Figure 3) [195].

Chronic rhinosinusitis is one of the most important and frequent comorbidities of severe asthma and is associated with worse outcomes and an increased risk of exacerbations in this type of patient [196,197]. The main histopathologic features of asthma, which include chronic eosinophilic inflammation, epithelial damage of the airway, and basement membrane thickening are shared and observed in sinonasal biopsies from patients with refractory CRSwNP [198].

**Figure 4:**
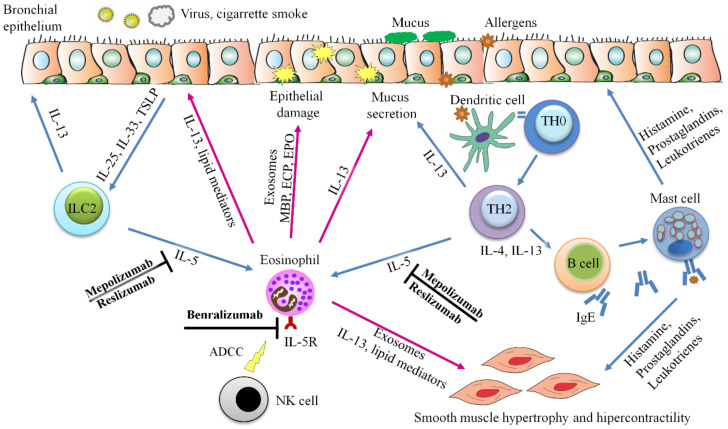
Role of eosinophils in the pathophysiology of asthma and biological drugs for its control. Eosinophils are crucial to the development and maintenance of the asthmatic symptoms, being attracted to the airways by two non-exclusive mechanisms. The first one involves activation of the innate immunity, where type 2 innate lymphoid cells (ILC2s) are activated by alarmins (IL-33, IL25 and TSLP) released after damage of airway epithelium and releasing Il-5, the main chemoattractant and inductor of eosinophils activity. Alternatively, or simultaneously, allergens traverse the epithelium and are recognized by dendritic cells and presented to T naïve helper cells (Th0), which are polarized to Th2 cells, secreting Il-4 and Il-13, stimulators of the release of allergen-specific IgE by B cells. IgE recognising the allergen is then able to induce the production of histamine, leukotrienes and prostaglandins by mast cells, being these molecules involved in smooth muscle hypertrophy and contraction. Besides, Th2 cells are also capable of releasing IL-5, which activates eosinophils in an allergen-mediated mechanism. Eosinophils stimulated by IL-5 releasing IL-13 and lipid mediators which activate the epithelium and induce mucus secretion. Also, eosinophils discharge exosomes, toxic proteins (ECP, MBP and EPO) and other mediators as ROS and NO capable of inducing epithelial damage. Exosomes, IL-13 and other lipid mediators released by eosinophils are also inductors of smooth muscle hypertrophy and contraction, highlighting the multiple-pathway pathophysiological role developed by these cells. Hence, biological drugs are indeed treatment options for controlling eosinophils adverse effects in asthma, such as mepolizumab and reslizumab which recognise IL-5 and block its binding over eosinophils, and benralizumab, a monoclonal antibody that binds to IL-5R and induces antibody-dependent cell-mediated cytotoxicity (ADCC) of natural killer (NK) cells over eosinophils.

Allergic asthma is characterized by inflammation of the airway causing airway obstruction, muscle hypertrophy, mucus secretion, and bronchial hyperresponsiveness, where eosinophils play a significant role in damaging the epithelium and orchestrating an immune response [199]. This link between eosinophils and asthma pathogenesis was first described in 1988 [200]. In eosinophilic asthma, called asthma T2, the number of eosinophils in the airways augment when the airway epithelium is exposed to an allergen or antigen, triggering the activation of several immunological cascades that drive eosinophils to the airways due to Th2 cytokines and chemoattractants [201] (Figure 1, Figure 3 and Figure 4).

In recent years, several studies have demonstrated that eosinophils themselves and the action through their exosomes (EVs that act as independent functional units and with a relevant role in intercellular communication) take part in the asthmatic process. By degranulation of eosinophils, toxic proteins such as EDN, ECP, EPO, MBP, cytokine-mediated activation (IL-5), and lipid mediators, such as cysteinyl leukotrienes (cysLTs) are released, contributing to the development and maintenance of the eosinophilic inflammation in the airways [202]. Cañas et al. [126], after defining the existence of exosomes produced by eosinophils [14], demonstrated that exosomes generated by eosinophils from asthmatic patients are able to promote eosinophil functions such as ROS or NO production, increasing their adhesion capacity and redirecting eosinophil migration, thereby contributing to an inflammatory response [126]. Moreover, the capacity of these asthmatic eosinophilic exosomes to contribute to the remodeling of structural components of the lungs and airways during the asthma process was established [127] (Figure 2 and Figure 3). So, eosinophils not only promote their own capacity for survival by autocrine secretion of IL-5, GM-CSF, and IL-3, explaining their survival at inflammatory sites [203,204]. This capacity also allows them to remain in the target tissue and exert their effects over a prolonged period of time.

In asthma disease, eosinophil count in the airways has been determined as a biomarker for this disease, and an increased eosinophil count in sputum is associated with poor asthma control, and more frequent exacerbations [205]. In this line, in a study of patients with intermittent or persistent eosinophilia in which sputum eosinophilia was measured longitudinally in a clinical setting, it was observed that a reduction (3.4-fold or 4.3%) of sputum eosinophilia predicted an improvement in asthma control whereas an increase (1.8-fold or 3.5%) of this eosinophilia was associated with a deterioration of control [206].

Blood eosinophil count is also a surrogate biomarker for type 2 airway inflammation. Several reports have shown that this parameter is capable of predicting severe asthma exacerbations, as well as responsiveness to inhaled corticosteroids (ICS) or novel biologic treatments for severe asthma, focused on IL-5 or eosinophils [207,208,209] (Figure 4). A prospective study performed by Zeiger et al. [210] showed that a blood eosinophil count of 0.4 × 109/L or greater was associated with a 1.55-fold increased risk of having 2 or more asthma exacerbations or asthma-related emergency department visits or hospitalizations over a 1-year period in patients with severe uncontrolled asthma with 12 years of age or older [210].

Interstitial lung disease (ILD) includes a large number of conditions that are characterized by inflammation or fibrosis of the pulmonary parenchyma [211]. ILD can occur due to a number of specific causes or may be classified as idiopathic interstitial pneumonia (IIP), a group of ILDs that lack a clear predisposing factor or underlying pathophysiology [212]. These diseases are characterized by an increased number of eosinophils in peripheral blood, in lung tissue, in sputum, in bronchoalveolar lavage fluid (BALF), or in all of them [213]. Therefore, cell populations recovered with bronchoalveolar lavage (BAL) may be important in predicting disease progression and response to therapy [214]. All these data bring to light the clear relevance of eosinophils in multiple diseases of varying origin with a wide range of symptoms in a highly complex scenario of relationships between different elements implicated in the pathogenesis. Added to all this is the heterogeneity of eosinophils.

## 7. Treatments Focused on Eosinophils

As with other pathologies, the goal of the treatment of eosinophilic diseases is to reduce the total eosinophil count and symptoms, achieving a clinical improvement and preventing the progression of the disease (Table 2).

Traditionally, systemic glucocorticoid treatment has been considered the first-line of therapeutic approach, though with the following exceptions: (1) eosinophilia due to a secondary cause such as helminth infection, drug hypersensitivity, or neoplasm; (2) patients with gene mutations in *PDGFRA* or *PDGFRB* in whom imatinib is the appropriate treatment; and (3) overlap syndromes that may respond to topical glucocorticoid treatment such as EGID [215]. As occurs in other leukocytes, corticoids cause apoptosis of eosinophils and inhibit the release of cytokines implicated in eosinophil survival [216]. Theophylline and antileukotrienes, both classical anti-asthmatic drugs, promote the death of eosinophils due to their anti-inflammatory effects [217] just like conventional immunosuppressors such as cyclophosphamide [218].

**Table 2:** Treatments to eosinophilic disorders.

Drug	Disease	Mechanism	Ref.
Glucocorticoid	Systemic inflammation	Apoptotic effects and inhibition of cytokines implicated in eosinophil survival	[216]
Theophylline	Asthma	Anti-inflammatory effects	[217]
Antileukotrienes	Asthma	Anti-inflammatory effects	[217]
Cyclophosphamide	Lymphoma	Immunosuppressor	[218]
Alemtezumab	Severe T cell neoplasms or inflammatory diseases	Anti-CD52	[218]
Levosimendan/destrosimendan	Commonly used to heart failure	Eosinophil proapoptotic effect in vitro	[219]
Bertilimumab	Pemphigus	Anti-eotaxin-1. Affect to eosinophil recruitment	[220]
Mepolizumab and reslizumab	Uncontrolled severe asthma	Anti-IL-5	[221,222,223,224]
Benralizumab	Uncontrolled severe asthma	Anti-IL-5R. Inhibits the growth, maturation, activation and survival of eosinophils through an antibody-dependent cytotoxicity mechanism	[225,226]
Omalizumab	Persistent severe allergic asthma	Anti-IgE	[227]
Dupilumab	Moderate-to-severe atopic dermatitis	Anti-IL-13	[228,229]
Tezepelumab	Severe asthma	Anti-TSLP	[230]

Biological treatments have emerged as promising therapeutic options in eosinophilic disorders. Some of them, such as alemtuzumab (anti-CD52), are based on the use of antibodies against surface molecules of eosinophils, although in this case therapy is more focused on severe T cell neoplasms or inflammatory diseases [218]. In this line, other promising strategies based upon the potentiation of eosinophil apoptosis are under research, including target molecules such as sialic acid-binding immunoglobulin-like lectin 8 (Siglec-8), although its expression in both regulatory and inflammatory eosinophils could jeopardize its development [164]. Other molecules under development are factors linked to the control of the cell cycle and regulatory elements of the intracellular ionic balance [216,217] such as calcium sensitizers, usually employed in heart failure and which, in vitro, exert proapoptotic effects on eosinophils, such as levosimendan or dextrosimendan [219]. Alternative approaches are based on substances that affect eosinophil recruitment through the mechanism of chemotaxis, such as anti-eotaxin-1 antibodies (bertilimumab), which have shown promising results [220].

In the field of respiratory diseases, biological treatments associated with type 2 inflammation in which eosinophils play a crucial role have emerged in recent years with a focus on the treatment of severe asthma. Eosinophils are one of the biomarkers of type 2 severe asthma phenotype [231], and like so, in other respiratory diseases such as chronic obstructive pulmonary disease (COPD), could act as a biomarker to identify patients with a particularly favorable response to inhaled corticosteroid/long-acting β-agonist therapy [232].

There are multiple commercially available novel drugs or drugs under development drugs that target eosinophils either directly or indirectly (Figure 4).

Mepolizumab and reslizumab are antibodies that target IL-5. Despite differences in age thresholds between them, both are licensed for use as add-on therapy in patients with uncontrolled asthma regardless of the use of medium or high dose of inhaled steroids; besides, these drugs are available for patients receiving or not oral corticosteroids who have peripheral blood eosinophilia (current eosinophils > 150/mm^3^ or eosinophils > 300/mm^3^ once in the previous year in the case of mepolizumab and eosinophils > 400/mm^3^ for the use of reslizumab). Both monoclonal antibodies prevent the binding of IL-5 to its receptor (IL-5R), preventing signaling in eosinophils. This blocking of IL-5 activity causes significant depletion of circulating eosinophils, reducing production, activation, and survival rate [221,222,223]. In the case of mepolizumab, its depletion effect on bronchial tissue eosinophilia is less marked, with a median reduction of 55% [224].

Benralizumab is a humanized, afucossylated monoclonal antibody that targets the α-chain of the IL-5 receptor (IL-5R) that is expressed by eosinophils and basophils. This drug is licensed as an add-on therapy in uncontrolled asthma with high doses of inhaled corticosteroids and with blood eosinophilia (eosinophils > 300/mmc) [225]. This drug inhibits the growth, maturation, activation, and survival of eosinophils, also causing ADCC, promoting eosinophil apoptosis through the delivery of pro-apoptotic factors [226].

Besides these anti-IL5 and anti-IL-5R drugs, other pharmaceutical options linked to biology and functions of eosinophils do also exist [228].

With different targets, omalizumab and dupilumab are two monoclonal antibodies used in asthmatic disease. IgE is the target of omalizumab. It was the first biologic therapy to be licensed for asthma, and the only biologic therapy to target IgE. Moreover, this anti-IgE humanized monoclonal antibody acts by significantly reducing the number of plasmacytoid dendritic cells (pDCs), which increase during asthma exacerbations [227].

Dupilumab is a humanized monoclonal antibody, that binds to the α subunit of IL-4 receptors. Its use is indicated in patients with moderate-to-severe asthma with a Th2-high phenotype (FeNO > 25 ppb or peripheral blood eosinophils >150/μL) receiving ICS and long-acting beta-agonists (LABA), with or without the need for oral corticosteroids [228,229].

Randomized clinical trials (RCTs) of severe eosinophilic asthma have demonstrated that the use of these type 2-targeting biologics (omalizumab, mepolizumab, reslizumab, benralizumab, and dupilumab) achieves a significant reduction in asthma exacerbations and oral corticosteroid use, improving lung function [233,234].

Recent research is focused on proinflammatory mediators such as prostaglandins and alarmins, including thymic stromal lymphopoietin (TSLP). Tezepelumab is a humanized monoclonal antibody, which targets the previously mentioned TSLP. This cytokine activates type 2 innate lymphoid cells (ILC2) which are able to trigger T2 immune response inflammation with an important role of eosinophils [230].

There is thus a vast arsenal of therapy approaches acting against eosinophils through direct or indirect pathways with different recommendations and evidence varying with disease and context.

One of the queries that arise with therapies that target eosinophils is whether this removal of eosinophils has deleterious consequences. However, studies on the safety of eosinophil-depleting drugs have not shown an increase of infection or neoplasia rate in these patients, is only associated with improvement on asthma without any secondary effects related [235]. Moreover, in subjects without eosinophils due to immunodeficiency or IgG eosinophil precursor destruction, there is no data confirming any abnormality. This is also observed in mouse models without eosinophils, characterized by normal health status in laboratory conditions [236]. All these data raise the question about the importance of these cells in physiological functions, emphasizing the redundancy of the immune system responses. Indeed, eosinophils have been described as important in several biological processes, but many of these studies were performed in mouse laboratory-controlled models and perhaps cannot be fully extrapolated to humans. The absence of a phenotype related to eosinophil depletion in humans suggests that eosinophils might not be essential in healthy maintenance, being its functions rather supportive. This is a future challenge for research in the eosinophil field.

## 8. Conclusions

Knowledge about eosinophils has experienced an outburst in the last few years. Recent advances have shown that they not only play a role in regulation in the context of parasitic infestations, but also play an important role at the homeostatic level, and in the development of type 2 diseases such as asthma or autoimmune pathologies, such as Crohn’s disease.

In recent years, this growth has been almost exponential with the arrival of the latest biological treatments that, directly or indirectly, target the eosinophil, revealing new features of this granulocyte, while putting into test the most traditional points of view for this granulocyte but also open questions about this intriguing cell.

## Data Availability

Not applicable.

## Abbreviations

ACE2	Angiotensin-converting enzyme 2
ADCC	Antibody-dependent cellular cytotoxicity
CCR3	CC-chemokine receptor 3
COPD	Chronic obstructive pulmonary disease
cysLTR	Cysteinyl leukotriene receptor
CP	Chronic pancreatitis
CRSsNP	Chronic rhinosinusitis without nasal polyps
CRSwNP	Chronic rhinosinusitis with nasal polyps
CRTH2	Prostaglandin D_2_ receptor 2
DC	Dendritic cell
DNA	Deoxyribonucleic acid
ECP	Eosinophil cationic protein
EDN	Eosinophil derived neurotoxin
EETs	Eosinophil extracellular traps
EGF	Epidermal growth factor
EGID	Eosinophilic gastrointestinal disease
EID	Eosinophil immune dysfunction
EoE	Eosinophilic esophagitis
EoP	Eosinophil progenitors
Eos	Eosinophils
EPX	Eosinophil peroxidase
EVs	Extracellular vesicles
FeNO	Fraction of exhaled nitric oxide
GI	Gastrointestinal
GM-CSF	Granulocyte-macrophage colony stimulating factor
hEos	Homeostatic eosinophils
hpf	High-power field
ICAM1	Intercellular adhesion molecule 1
ICS	Inhaled corticosteroids
ICU	Intensive care unit
iEos	Inflammatory eosinophils
IFN	Interferon
IL-5	Interleukin-5
ILC2	Type 2 innate lymphoid cells
LABA	Long-acting beta agonists
MBP	Major basic protein
MHC	Major histocompatibility complex
miRNA	MicroRNA
NO	Nitric oxide
pDCs	Plasmacytoid dendritic cells
PDGFR	Platelet-derived growth factor receptor
RCTs	Randomized clinical trials
RNA	Ribonucleic acid
ROS	Reactive oxygen species
RSV	Respiratory syncytial virus
RT-PCR	Reverse transcription polymerase chain reaction
RTC	Randomized clinical trial
SARS-CoV-2	Severe acute respiratory syndrome coronavirus 2
Siglec-8	Sialic acid-binding immunoglobulin-like lectin 8
SNPs	Single nucleotide polymorphisms
TGF	Transforming growth factor
TNF-α	Tumor necrosis factor α
TLR	Toll-like receptor
TSLP	Thymic stromal lymphopoietin

## References

[1] Kay A.B. (2015). The early history of the eosinophil. Clin. Exp. Allergy.

[2] Gleich G.J., Adolphson C.R. (1986). The Eosinophilic Leukocyte: Structure and Function. Adv. Immunol..

[3] Lacy P., Rosenberg H.F., Walsh G.M. (2014). Eosinophil overview: Structure, biological properties, and key functions. Methods Mol. Biol..

[4] Dorosz A., Grosicki M., Dybas J., Matuszyk E., Rodewald M., Meyer T., Popp J., Malek K., Baranska M. (2020). Eosinophils and Neutrophils-Molecular Differences Revealed by Spontaneous Raman, CARS and Fluorescence Microscopy. Cells.

[5] Klion A.D., Ackerman S.J., Bochner B.S. (2020). Contributions of Eosinophils to Human Health and Disease. Annu. Rev. Pathol. Mech. Dis..

[6] Weller P.F., Spencer L.A. (2017). Functions of tissue-resident eosinophils. Nat. Rev. Immunol..

[7] Acharya K.R., Ackerman S.J. (2014). Eosinophil granule proteins: Form and function. J. Biol. Chem..

[8] Plager D.A., Loegering D.A., Checkel J.L., Tang J., Kephart G.M., Caffes P.L., Adolphson C.R., Ohnuki L.E., Gleich G.J. (2006). Major Basic Protein Homolog (MBP2): A Specific Human Eosinophil Marker. J. Immunol..

[9] Davoine F., Lacy P. (2014). Eosinophil cytokines, chemokines, and growth factors: Emerging roles in immunity. Front. Immunol..

[10] Woschnagg C., Rubin J., Venge P. (2009). Eosinophil Cationic Protein (ECP) Is Processed during Secretion. J. Immunol..

[11] Melo R.C.N., Weller P.F. (2018). Contemporary understanding of the secretory granules in human eosinophils. J. Leukoc. Biol..

[12] Ueki S., Miyabe Y., Yohei Y., Fukuchi M., Hirokawa M., Spencer L.A., Weller P.F. (2019). Charcot-Leyden Crystals in Eosinophilic Inflammation: Active Cytolysis Leads to Crystal Formation. Curr. Allergy Asthma Rep..

[13] Melo R.C.N., Weller P.F. (2014). Unraveling the complexity of lipid body organelles in human eosinophils. J. Leukoc. Biol..

[14] Mazzeo C., Cañas J.A., Zafra M.P., Rojas Marco A., Fernández-Nieto M., Sanz V., Mittelbrunn M., Izquierdo M., Baixaulli F., Sastre J. (2015). Exosome secretion by eosinophils: A possible role in asthma pathogenesis. J. Allergy Clin. Immunol..

[15] O’Sullivan J.A., Bochner B.S. (2018). Eosinophils and eosinophil-associated diseases: An update. J. Allergy Clin. Immunol..

[16] Mori Y., Iwasaki H., Kohno K., Yoshimoto G., Kikushige Y., Okeda A., Uike N., Niiro H., Takenaka K., Nagafuji K. (2009). Identification of the human eosinophil lineage-committed progenitor: Revision of phenotypic definition of the human common myeloid progenitor. J. Exp. Med..

[17] Bossios A., Rådinger M. (2014). CD34+ eosinophil-lineage-committed cells in the mouse lung. Methods Mol. Biol..

[18] Stirling R.G., Van Rensen E.L.J., Barnes P.J., Chung K.F. (2001). Interleukin-5 induces CD34+ eosinophil progenitor mobilization and eosinophil CCR3 expression in asthma. Am. J. Respir. Crit. Care Med..

[19] Robinson D.S., Damia R., Zeibecoglou K., Molet S., North J., Yamada T., Kay A.B., Hamid Q. (1999). CD34+/interleukin-5Rα messenger RNA+ cells in the bronchial mucosa in asthma: Potential airway eosinophil progenitors. Am. J. Respir. Cell Mol. Biol..

[20] Fulkerson P.C. (2017). Transcription factors in eosinophil development and as therapeutic targets. Front. Med..

[21] Bouffi C., Kartashov A.V., Schollaert K.L., Chen X., Bacon W.C., Weirauch M.T., Barski A., Fulkerson P.C. (2015). Transcription Factor Repertoire of Homeostatic Eosinophilopoiesis. J. Immunol..

[22] Yu C., Cantor A.B., Yang H., Browne C., Wells R.A., Fujiwara Y., Orkin S.H. (2002). Targeted deletion of a high-affinity GATA-binding site in the GATA-1 promoter leads to selective loss of the eosinophil lineage in vivo. J. Exp. Med..

[23] Drissen R., Thongjuea S., Theilgaard-Mönch K., Nerlov C. (2019). Identification of two distinct pathways of human myelopoiesis. Sci. Immunol..

[24] Pellin D., Loperfido M., Baricordi C., Wolock S.L., Montepeloso A., Weinberg O.K., Biffi A., Klein A.M., Biasco L. (2019). A comprehensive single cell transcriptional landscape of human hematopoietic progenitors. Nat. Commun..

[25] Weller P.F. (1992). Cytokine regulation of eosinophil function. Clin. Immunol. Immunopathol..

[26] Dougan M., Dranoff G., Dougan S.K. (2019). GM-CSF, IL-3, and IL-5 Family of Cytokines: Regulators of Inflammation. Immunity.

[27] Rothenberg M.E., Hogan S.P. (2006). The eosinophil. Annu. Rev. Immunol..

[28] Pelaia C., Paoletti G., Puggioni F., Racca F., Pelaia G., Canonica G.W., Heffler E. (2019). Interleukin-5 in the Pathophysiology of Severe Asthma. Front. Physiol..

[29] Mortaz E., Amani S., Mumby S., Adcock I.M., Movassaghi M., Folkerts J., Garssen J., Folkerts G. (2018). Role of mast cells and Type 2 Innate Lymphoid (ILC2) cells in lung transplantation. J. Immunol. Res..

[30] Kim H.J., Jung Y. (2020). The Emerging Role of Eosinophils as Multifunctional Leukocytes in Health and Disease. Immune Netw..

[31] Nakagome K., Nagata M. (2020). Possible mechanisms of eosinophil accumulation in eosinophilic pneumonia. Biomolecules.

[32] Powell W.S., Rokach J. (2013). The eosinophil chemoattractant 5-oxo-ETE and the OXE receptor. Prog. Lipid Res..

[33] Esnault S., Kelly E.A. (2016). Essential mechanisms of differential activation of eosinophils by IL-3 compared to GM-CSF and IL-5. Crit. Rev. Immunol..

[34] Schwartz C., Willebrand R., Huber S., Rupec R.A., Wu D., Locksley R., Voehringer D. (2015). Eosinophil-specific deletion of IκBα in mice reveals a critical role of NF-κB-induced Bcl-xLfor inhibition of apoptosis. Blood.

[35] Mitchell P.D., O’Byrne P.M. (2017). Epithelial-Derived Cytokines in Asthma. Chest.

[36] Johnston L.K., Hsu C.-L., Krier-Burris R.A., Chhiba K.D., Chien K.B., McKenzie A., Berdnikovs S., Bryce P.J. (2016). IL-33 Precedes IL-5 in Regulating Eosinophil Commitment and Is Required for Eosinophil Homeostasis. J. Immunol..

[37] Heffler E., Allegra A., Pioggia G., Picardi G., Musolino C., Gangemi S. (2017). MicroRNA profiling in asthma: Potential biomarkers and therapeutic targets. Am. J. Respir. Cell Mol. Biol..

[38] Bochner B.S. (2015). Novel Therapies for Eosinophilic Disorders. Immunol. Allergy Clin. N. Am..

[39] Takatsu K. (2011). Interleukin-5 and IL-5 receptor in health and diseases. Proc. Jpn. Acad. Ser. B Phys. Biol. Sci..

[40] Lamkhioued B., Abdelilah S.G., Hamid Q., Mansour N., Delespesse G., Renzi P.M. (2003). The CCR3 Receptor Is Involved in Eosinophil Differentiation and Is Up-Regulated by Th2 Cytokines in CD34 + Progenitor Cells. J. Immunol..

[41] Youngblood B.A., Brock E.C., Leung J., Falahati R., Bochner B.S., Rasmussen H.S., Peterson K., Bebbington C., Tomasevic N. (2019). Siglec-8 antibody reduces eosinophils and mast cells in a transgenic mouse model of eosinophilic gastroenteritis. JCI Insight.

[42] Legrand F., Tomasevic N., Simakova O., Lee C.C.R., Wang Z., Raffeld M., Makiya M.A., Palath V., Leung J., Baer M. (2014). The eosinophil surface receptor epidermal growth factor-like module containing mucin-like hormone receptor 1 (EMR1): A novel therapeutic target for eosinophilic disorders. J. Allergy Clin. Immunol..

[43] Sastre B., Rodrigo-Muñoz J.M., Garcia-Sanchez D.A., Cañas J.A., Del Pozo V. (2018). Eosinophils: Old players in a new game. J. Investig. Allergol. Clin. Immunol..

[44] Yoon J., Um H.N., Jang J., Bae Y.A., Park W.J., Kim H.J., Yoon M.S., Chung I.Y., Jung Y.J. (2019). Eosinophil Activation by Toll-Like Receptor 4 Ligands Regulates Macrophage Polarization. Front. Cell Dev. Biol..

[45] Zustakova M., Kratochvilova L., Slama P. (2020). Apoptosis of eosinophil granulocytes. Biology.

[46] Kovalszki A., Weller P.F. (2016). Eosinophilia. Prim. Care Clin. Off. Pract..

[47] Kita H. (2013). Eosinophils: Multifunctional and distinctive properties. Int. Arch. Allergy Immunol..

[48] Gieseck R.L., Wilson M.S., Wynn T.A. (2018). Type 2 immunity in tissue repair and fibrosis. Nat. Rev. Immunol..

[49] Doran E., Cai F., Holweg C.T.J., Wong K., Brumm J., Arron J.R. (2017). Interleukin-13 in Asthma and Other Eosinophilic Disorders. Front. Med..

[50] Percopo C.M., Dyer K.D., Ochkur S.I., Luo J.L., Fischer E.R., Lee J.J., Lee N.A., Domachowske J.B., Rosenberg H.F. (2014). Activated mouse eosinophils protect against lethal respiratory virus infection. Blood.

[51] Huang L., Gebreselassie N.G., Gagliardo L.F., Ruyechan M.C., Luber K.L., Lee N.A., Lee J.J., Appleton J.A. (2015). Eosinophils Mediate Protective Immunity against Secondary Nematode Infection. J. Immunol..

[52] Huang L., Appleton J.A. (2016). Eosinophils in Helminth Infection: Defenders and Dupes. Trends Parasitol..

[53] Scepek S., Moqbel R., Lindau M. (1994). Compound exocytosis and cumulative degranulation by eosinophils and their role in parasite killing. Parasitol. Today.

[54] Melo R.C.N., Perez S.A.C., Spencer L.A., Dvorak A.M., Weller P.F. (2005). Intragranular vesiculotubular compartments are involved in piecemeal degranulation by activated human eosinophils. Traffic.

[55] Carmo L.A.S., Bonjour K., Ueki S., Neves J.S., Liu L., Spencer L.A., Dvorak A.M., Weller P.F., Melo R.C.N. (2016). CD63 is tightly associated with intracellular, secretory events chaperoning piecemeal degranulation and compound exocytosis in human eosinophils. J. Leukoc. Biol..

[56] Dias F.F., Amaral K.B., Malta K.K., Silva T.P., Rodrigues G.S.C., Rosa F.M., Rodrigues G.O.L., Costa V.V., Chiarini-Garcia H., Weller P.F. (2018). Identification of Piecemeal Degranulation and Vesicular Transport of MBP-1 in Liver-Infiltrating Mouse Eosinophils During Acute Experimental Schistosoma mansoni Infection. Front. Immunol..

[57] Spencer L.A., Melo R.C.N., Perez S.A.C., Bafford S.P., Dvorak A.M., Weller P.F. (2006). Cytokine receptor-mediated trafficking of preformed IL-4 in eosinophils identifies an innate immune mechanism of cytokine secretion. Proc. Natl. Acad. Sci. USA.

[58] Neves J.S., Radke A.L., Weller P.F. (2010). Cysteinyl leukotrienes acting via granule membrane-expressed receptors elicit secretion from within cell-free human eosinophil granules. J. Allergy Clin. Immunol..

[59] Adu B., Dodoo D., Adukpo S., Gyan B.A., Hedley P.L., Goka B., Adjei G.O., Larsen S.O., Christiansen M., Theisen M. (2011). Polymorphisms in the RNASE3 gene are associated with susceptibility to cerebral malaria in Ghanaian children. PLoS ONE.

[60] Diop G., Derbois C., Loucoubar C., Mbengue B., Ndao B.N., Thiam F., Thiam A., Ndiaye R., Dieye Y., Olaso R. (2018). Genetic variants of RNASE3 (ECP) and susceptibility to severe malaria in Senegalese population. Malar. J..

[61] Fabre V., Beiting D.P., Bliss S.K., Gebreselassie N.G., Gagliardo L.F., Lee N.A., Lee J.J., Appleton J.A. (2009). Eosinophil Deficiency Compromises Parasite Survival in Chronic Nematode Infection. J. Immunol..

[62] Huang L., Gebreselassie N.G., Gagliardo L.F., Ruyechan M.C., Lee N.A., Lee J.J., Appleton J.A. (2014). Eosinophil-Derived IL-10 Supports Chronic Nematode Infection. J. Immunol..

[63] Radonjic-Hoesli S., Wang X., de Graauw E., Stoeckle C., Styp-Rekowska B., Hlushchuk R., Simon D., Spaeth P.J., Yousefi S., Simon H.U. (2017). Adhesion-induced eosinophil cytolysis requires the receptor-interacting protein kinase 3 (RIPK3)–mixed lineage kinase-like (MLKL) signaling pathway, which is counterregulated by autophagy. J. Allergy Clin. Immunol..

[64] Neves J.S., Perez S.A.C., Spencer L.A., Melo R.C.N., Reynolds L., Ghiran I., Mahmudi-Azer S., Odemuyiwa S.O., Dvorak A.M., Moqbel R. (2008). Eosinophil granules function extracellularly as receptor-mediated secretory organelles. Proc. Natl. Acad. Sci. USA.

[65] Ueki S., Melo R.C.N., Ghiran I., Spencer L.A., Dvorak A.M., Weller P.F. (2013). Eosinophil extracellular DNA trap cell death mediates lytic release of free secretion-competent eosinophil granules in humans. Blood.

[66] Yousefi S., Gold J.A., Andina N., Lee J.J., Kelly A.M., Kozlowski E., Schmid I., Straumann A., Reichenbach J., Gleich G.J. (2008). Catapult-like release of mitochondrial DNA by eosinophils contributes to antibacterial defense. Nat. Med..

[67] Muniz V.S., Silva J.C., Braga Y.A.V., Melo R.C.N., Ueki S., Takeda M., Hebisawa A., Asano K., Figueiredo R.T., Neves J.S. (2018). Eosinophils release extracellular DNA traps in response to Aspergillus fumigatus. J. Allergy Clin. Immunol..

[68] Gevaert E., Zhang N., Krysko O., Lan F., Holtappels G., De Ruyck N., Nauwynck H., Yousefi S., Simon H.U., Bachert C. (2017). Extracellular eosinophilic traps in association with Staphylococcus aureus at the site of epithelial barrier defects in patients with severe airway inflammation. J. Allergy Clin. Immunol..

[69] Rosenberg H.F., Masterson J.C., Furuta G.T. (2016). Eosinophils, probiotics, and the microbiome. J. Leukoc. Biol..

[70] Svensson L., Wennerås C. (2005). Human eosinophils selectively recognize and become activated by bacteria belonging to different taxonomic groups. Microbes Infect..

[71] Linch S.N., Kelly A.M., Danielson E.T., Pero R., Lee J.J., Gold J.A. (2009). Mouse eosinophils possess potent antibacterial properties in vivo. Infect. Immun..

[72] Torrent M., de la Torre B.G., Nogués V.M., Andreu D., Boix E. (2009). Bactericidal and membrane disruption activities of the eosinophil cationic protein are largely retained in an N-terminal fragment. Biochem. J..

[73] Dworski R., Simon H.U., Hoskins A., Yousefi S. (2011). Eosinophil and neutrophil extracellular DNA traps in human allergic asthmatic airways. J. Allergy Clin. Immunol..

[74] Choi Y., Le Pham D., Lee D.H., Lee S.H., Kim S.H., Park H.S. (2018). Biological function of eosinophil extracellular traps in patients with severe eosinophilic asthma. Exp. Mol. Med..

[75] Simon D., Radonjic-Hösli S., Straumann A., Yousefi S., Simon H.U. (2015). Active eosinophilic esophagitis is characterized by epithelial barrier defects and eosinophil extracellular trap formation. Allergy Eur. J. Allergy Clin. Immunol..

[76] Melo R.C.N., Wang H., Silva T.P., Imoto Y., Fujieda S., Fukuchi M., Miyabe Y., Hirokawa M., Ueki S., Weller P.F. (2020). Galectin-10, the protein that forms Charcot-Leyden crystals, is not stored in granules but resides in the peripheral cytoplasm of human eosinophils. J. Leukoc. Biol..

[77] Prince L.R., Graham K.J., Connolly J., Anwar S., Ridley R., Sabroe I., Foster S.J., Whyte M.K.B. (2012). Staphylococcus aureus induces eosinophil cell death mediated by α-Hemolysin. PLoS ONE.

[78] Li J., Boix E. (2021). Host Defence RNases as Antiviral Agents against Enveloped Single Stranded RNA Viruses. Virulence.

[79] Phipps S., En Lam C., Mahalingam S., Newhouse M., Ramirez R., Rosenberg H.F., Foster P.S., Matthaei K.I. (2007). Eosinophils contribute to innate antiviral immunity and promote clearance of respiratory syncytial virus. Blood.

[80] Samarasinghe A.E., Melo R.C.N., Duan S., LeMessurier K.S., Liedmann S., Surman S.L., Lee J.J., Hurwitz J.L., Thomas P.G., McCullers J.A. (2017). Eosinophils Promote Antiviral Immunity in Mice Infected with Influenza A Virus. J. Immunol..

[81] Drake M.G., Bivins-Smith E.R., Proskocil B.J., Nie Z., Scott G.D., Lee J.J., Lee N.A., Fryer A.D., Jacoby D.B. (2016). Human and mouse eosinophils have antiviral activity against parainfluenza virus. Am. J. Respir. Cell Mol. Biol..

[82] Ma M., Redes J.L., Percopo C.M., Druey K.M., Rosenberg H.F. (2018). Alternaria alternata challenge at the nasal mucosa results in eosinophilic inflammation and increased susceptibility to influenza virus infection. Clin. Exp. Allergy.

[83] Flores-Torres A.S., Salinas-Carmona M.C., Salinas E., Rosas-Taraco A.G. (2019). Eosinophils-Respiratory Viruses. Viral Immunol..

[84] Rodrigo-Muñoz J., Sastre B., Cañas J., Gil-Martínez M., Redondo N., del Pozo V. (2020). Eosinophil Response Against Classical and Emerging Respiratory Viruses: COVID-19. J. Investig. Allergol. Clin. Immunol..

[85] Nair A.P., Soliman A., Al Masalamani M.A., De Sanctis V., Nashwan A.J., Sasi S., Ali E.A., Hassan O.A., Iqbal F.M., Yassin M.A. (2020). Clinical outcome of eosinophilia in patients with covid-19: A controlled study. Acta Biomed..

[86] Ferastraoaru D., Hudes G., Jerschow E., Jariwala S., Karagic M., de Vos G., Rosenstreich D., Ramesh M. (2021). Eosinophilia in Asthma Patients Is Protective Against Severe COVID-19 Illness. J. Allergy Clin. Immunol. Pract..

[87] Mateos González M., Sierra Gonzalo E., Casado Lopez I., Arnalich Fernández F., Beato Pérez J.L., Monge Monge D., Vargas Núñez J.A., García Fenoll R., Suárez Fernández C., Freire Castro S.J. (2021). The Prognostic Value of Eosinophil Recovery in COVID-19: A Multicentre, Retrospective Cohort Study on Patients Hospitalised in Spanish Hospitals. J. Clin. Med..

[88] Liu F., Xu A., Zhang Y., Xuan W., Yan T., Pan K., Yu W., Zhang J. (2020). Patients of COVID-19 may benefit from sustained Lopinavir-combined regimen and the increase of Eosinophil may predict the outcome of COVID-19 progression. Int. J. Infect. Dis..

[89] Glickman J.W., Pavel A.B., Guttman-Yassky E., Miller R.L. (2021). The role of circulating eosinophils on COVID-19 mortality varies by race/ethnicity. J. Allergy Clin. Immunol..

[90] Xie G., Ding F., Han L., Yin D., Lu H., Zhang M. (2021). The role of peripheral blood eosinophil counts in COVID-19 patients. Allergy Eur. J. Allergy Clin. Immunol..

[91] Yang J., Zhao X., Liu X., Sun W., Zhou L., Wang Y., Sui H. (2020). Clinical Characteristics and Eosinophils in Young SARS-CoV-2-Positive Chinese Travelers Returning to Shanghai. Front. Public Health.

[92] Huang J., Zhang Z., Liu S., Gong C., Chen L., Ai G., Zhu X., Zhang C., Li D. (2020). Absolute Eosinophil Count Predicts Intensive Care Unit Transfer Among Elderly COVID-19 Patients From General Isolation Wards. Front. Med..

[93] Georgakopoulou V.E., Garmpis N., Damaskos C., Valsami S., Dimitroulis D., Diamantis E., Farmaki P., Papageorgiou C.V., Makrodimitri S., Gravvanis N. (2021). The impact of peripheral eosinophil counts and eosinophil to lymphocyte ratio (ELR) in the clinical course of covid-19 patients: A retrospective study. In Vivo.

[94] Tan Y., Zhou J., Zhou Q., Hu L., Long Y. (2021). Role of eosinophils in the diagnosis and prognostic evaluation of COVID-19. J. Med. Virol..

[95] Mu T., Yi Z., Wang M., Wang J., Zhang C., Chen H., Bai M., Jiang L., Zhang Y. (2021). Expression of eosinophil in peripheral blood of patients with COVID-19 and its clinical significance. J. Clin. Lab. Anal..

[96] Yan B., Yang J., Xie Y., Tang X. (2021). Relationship between blood eosinophil levels and COVID-19 mortality. World Allergy Organ. J..

[97] Lucas C., Wong P., Klein J., Castro T.B.R., Silva J., Sundaram M., Ellingson M.K., Mao T., Oh J.E., Israelow B. (2020). Longitudinal analyses reveal immunological misfiring in severe COVID-19. Nature.

[98] Roncati L., Nasillo V., Lusenti B., Riva G. (2020). Signals of Th2 immune response from COVID-19 patients requiring intensive care. Ann. Hematol..

[99] Rodriguez L., Pekkarinen P.T., Lakshmikanth T., Tan Z., Consiglio C.R., Pou C., Chen Y., Mugabo C.H., Nguyen N.A., Nowlan K. (2020). Systems-Level Immunomonitoring from Acute to Recovery Phase of Severe COVID-19. Cell Rep. Med..

[100] Pala D., Pistis M. (2021). Anti-IL5 Drugs in COVID-19 Patients: Role of Eosinophils in SARS-CoV-2-Induced Immunopathology. Front. Pharmacol..

[101] Gan J., Li J., Li S., Yang C. (2020). Leucocyte Subsets Effectively Predict the Clinical Outcome of Patients With COVID-19 Pneumonia: A Retrospective Case-Control Study. Front. Public Health.

[102] Soni M. (2020). Evaluation of eosinopenia as a diagnostic and prognostic indicator in COVID-19 infection. Int. J. Lab. Hematol..

[103] Djangang N.N., Peluso L., Talamonti M., Izzi A., Gevenois P.A., Garufi A., Goffard J.C., Henrard S., Severgnini P., Vincent J.L. (2020). Eosinopenia in COVID-19 patients: A retrospective analysis. Microorganisms.

[104] Vial R., Gully M., Bobot M., Scarfoglière V., Brunet P., Bouchouareb D., Duval A., Zino H.-O., Faraut J., Jehel O. (2020). Triage of Patients Suspected of COVID-19 in Chronic Hemodialysis: Eosinophil Count Differentiates Low and High Suspicion of COVID-19. J. Clin. Med..

[105] Outh R., Boutin C., Gueudet P., Suzuki M., Saada M., Aumaître H. (2021). Eosinopenia <100/μL as a marker of active COVID-19: An observational prospective study. J. Microbiol. Immunol. Infect..

[106] Lippi G., Sanchis-Gomar F., Henry B.M. (2021). Response to: Eosinophil count in coronavirus disease 2019: More doubts than answers. QJM.

[107] Kevadiya B.D., Machhi J., Herskovitz J., Oleynikov M.D., Blomberg W.R., Bajwa N., Soni D., Das S., Hasan M., Patel M. (2021). Diagnostics for SARS-CoV-2 infections. Nat. Mater..

[108] Barroso B., Valverde-Monge M., Cañas Jose A., Rodrigo-Muñoz J.M., Gonzalez-Cano B., Villalobos-Violan V., Betancor D., Gomez-Cardeñosa A., Vallejo-Chamorro G., Baptista-Serna L. (2020). Prevalence, characteristics, and outcome of asthmatic patients with type 2 diseases in hospitalized patients with COVID-19 in Madrid, Spain. J. Investig. Allergol. Clin. Immunol..

[109] Jackson D.J., Busse W.W., Bacharier L.B., Kattan M., O’Connor G.T., Wood R.A., Visness C.M., Durham S.R., Larson D., Esnault S. (2020). Association of respiratory allergy, asthma, and expression of the SARS-CoV-2 receptor ACE2. J. Allergy Clin. Immunol..

[110] Camiolo M., Gauthier M., Kaminski N., Ray A., Wenzel S.E. (2020). Expression of SARS-CoV-2 receptor ACE2 and coincident host response signature varies by asthma inflammatory phenotype. J. Allergy Clin. Immunol..

[111] Hanon S., Brusselle G., Deschampheleire M., Louis R., Michils A., Peché R., Pilette C., Rummens P., Schuermans D., Simonis H. (2020). COVID-19 and biologics in severe asthma: Data from the Belgian Severe Asthma Registry. Eur. Respir. J..

[112] Rial M.J., Valverde M., del Pozo V., González-Barcala F.J., Martínez-Rivera C., Muñoz X., Olaguibel J.M., Plaza V., Curto E., Quirce S. (2021). Clinical characteristics in 545 patients with severe asthma on biological treatment during the COVID-19 outbreak. J. Allergy Clin. Immunol. Pract..

[113] Heffler E., Detoraki A., Contoli M., Papi A., Paoletti G., Malipiero G., Brussino L., Crimi C., Morrone D., Padovani M. (2021). COVID-19 in Severe Asthma Network in Italy (SANI) patients: Clinical features, impact of comorbidities and treatments. Allergy.

[114] Eger K., Hashimoto S., Braunstahl G.J., Ten Brinke A., Patberg K.W., Beukert A., Smeenk F., van der Sar–van der Brugge S., Weersink E.J.M., Bel E.H. (2021). Poor outcome of SARS-CoV-2 infection in patients with severe asthma on biologic therapy. Respir. Med..

[115] García-Moguel I., Díaz Campos R., Alonso Charterina S., Fernández Rodríguez C., Fernández Crespo J. (2020). COVID-19, severe asthma, and biologics. Ann. Allergy, Asthma Immunol..

[116] Renner A., Marth K., Patocka K., Idzko M., Pohl W. (2020). COVID-19 in two severe asthmatics receiving benralizumab: Busting the eosinophilia myth. ERJ Open Res..

[117] Renner A., Marth K., Patocka K., Pohl W. (2020). COVID-19 in a severe eosinophilic asthmatic receiving benralizumab–a case study. J. Asthma.

[118] Roca E., Ventura L., Zattra C.M., Lombardi C. (2021). EOSINOPENIA: An early, effective and relevant COVID-19 biomarker?. QJM.

[119] Bolles M., Deming D., Long K., Agnihothram S., Whitmore A., Ferris M., Funkhouser W., Gralinski L., Totura A., Heise M. (2011). A Double-Inactivated Severe Acute Respiratory Syndrome Coronavirus Vaccine Provides Incomplete Protection in Mice and Induces Increased Eosinophilic Proinflammatory Pulmonary Response upon Challenge. J. Virol..

[120] Tseng C.T., Sbrana E., Iwata-Yoshikawa N., Newman P.C., Garron T., Atmar R.L., Peters C.J., Couch R.B. (2012). Immunization with SARS coronavirus vaccines leads to pulmonary immunopathology on challenge with the SARS virus. PLoS ONE.

[121] Honda-Okubo Y., Barnard D., Ong C.H., Peng B.-H., Tseng C.-T.K., Petrovsky N. (2015). Severe Acute Respiratory Syndrome-Associated Coronavirus Vaccines Formulated with Delta Inulin Adjuvants Provide Enhanced Protection while Ameliorating Lung Eosinophilic Immunopathology. J. Virol..

[122] Iwata-Yoshikawa N., Uda A., Suzuki T., Tsunetsugu-Yokota Y., Sato Y., Morikawa S., Tashiro M., Sata T., Hasegawa H., Nagata N. (2014). Effects of Toll-Like Receptor Stimulation on Eosinophilic Infiltration in Lungs of BALB/c Mice Immunized with UV-Inactivated Severe Acute Respiratory Syndrome-Related Coronavirus Vaccine. J. Virol..

[123] Rosenberg H.F., Foster P.S. (2021). Eosinophils and COVID-19: Diagnosis, prognosis, and vaccination strategies. Semin. Immunopathol..

[124] Kubo H. (2018). Extracellular Vesicles in Lung Disease. Chest.

[125] Akuthota P., Carmo L.A.S., Bonjour K., Murphy R.O., Silva T.P., Gamalier J.P., Capron K.L., Tigges J., Toxavidis V., Camacho V. (2016). Extracellular Microvesicle Production by Human Eosinophils Activated by “Inflammatory” Stimuli. Front. Cell Dev. Biol..

[126] Cañas J.A., Sastre B., Mazzeo C., Fernández-Nieto M., Rodrigo-Muñoz J.M., González-Guerra A., Izquierdo M., Barranco P., Quirce S., Sastre J. (2017). Exosomes from eosinophils autoregulate and promote eosinophil functions. J. Leukoc. Biol..

[127] Cañas J.A., Sastre B., Rodrigo-Muñoz J.M., Fernández-Nieto M., Barranco P., Quirce S., Sastre J., del Pozo V. (2018). Eosinophil-derived exosomes contribute to asthma remodelling by activating structural lung cells. Clin. Exp. Allergy.

[128] Rodrigo-Muñoz J.M., Cañas J.A., Sastre B., Rego N., Greif G., Rial M., Mínguez P., Mahíllo-Fernández I., Fernández-Nieto M., Mora I. (2019). Asthma diagnosis using integrated analysis of eosinophil microRNAs. Allergy Eur. J. Allergy Clin. Immunol..

[129] Rodrigo-Muñoz J.M., Rial M.J., Sastre B., Cañas J.A., Mahíllo-Fernández I., Quirce S., Sastre J., Cosío B.G., Del Pozo V. (2019). Circulating miRNAs as diagnostic tool for discrimination of respiratory disease: Asthma, asthma-chronic obstructive pulmonary disease (COPD) overlap and COPD. Allergy.

[130] Jacobsen E.A., Jackson D.J., Heffler E., Mathur S.K., Bredenoord A.J., Pavord I.D., Akuthota P., Roufosse F., Rothenberg M.E. (2021). Eosinophil Knockout Humans: Uncovering the Role of Eosinophils Through Eosinophil-Directed Biological Therapies. Annu. Rev. Immunol..

[131] Nussbaum J.C., Van Dyken S.J., Von Moltke J., Cheng L.E., Mohapatra A., Molofsky A.B., Thornton E.E., Krummel M.F., Chawla A., Liang H.E. (2013). Type 2 innate lymphoid cells control eosinophil homeostasis. Nature.

[132] Jung Y., Wen T., Mingler M.K., Caldwell J.M., Wang Y.H., Chaplin D.D., Lee E.H., Jang M.H., Woo S.Y., Seoh J.Y. (2015). IL-1β in eosinophil-mediated small intestinal homeostasis and IgA production. Mucosal Immunol..

[133] Sugawara R., Lee E.J., Jang M.S., Jeun E.J., Hong C.P., Kim J.H., Park A., Yun C.H., Hong S.W., Kim Y.M. (2016). Small intestinal eosinophils regulate Th17 cells by producing IL-1 receptor antagonist. J. Exp. Med..

[134] Chu V.T., Beller A., Rausch S., Strandmark J., Zänker M., Arbach O., Kruglov A., Berek C. (2014). Eosinophils promote generation and maintenance of immunoglobulin-A-expressing plasma cells and contribute to gut immune homeostasis. Immunity.

[135] Chen H.H., Sun A.H., Ojcius D.M., Hu W.L., Ge Y.M., Lin X., Li L.J., Pan J.P., Yan J. (2015). Eosinophils from murine lamina propria induce differentiation of naïve T cells into regulatory T cells via TGF-β1 and retinoic acid. PLoS ONE.

[136] Gomez Torrijos E., Gonzalez-Mendiola R., Alvarado M., Avila R., Prieto-Garcia A., Valbuena T., Borja J., Infante S., Lopez M.P., Marchan E. (2018). Eosinophilic Esophagitis: Review and Update. Front. Med..

[137] Gouon-Evans V., Lin E.Y., Pollard J.W. (2002). Requirement of macrophages and eosinophils and their cytokines/chemokines for mammary gland development. Breast Cancer Res..

[138] Aupperlee M.D., Zhao Y., Tan Y.S., Leipprandt J.R., Bennett J., Haslam S.Z., Schwartz R.C. (2014). Epidermal growth factor receptor (EGFR) signaling is a key mediator of hormone-induced leukocyte infiltration in the pubertal female mammary gland. Endocrinology.

[139] Vicetti Miguel R.D., Quispe Calla N.E., Dixon D., Foster R.A., Gambotto A., Pavelko S.D., Hall-Stoodley L., Cherpes T.L. (2017). IL-4–secreting eosinophils promote endometrial stromal cell proliferation and prevent Chlamydia-induced upper genital tract damage. Proc. Natl. Acad. Sci. USA.

[140] Wu D., Molofsky A.B., Liang H.E., Ricardo-Gonzalez R.R., Jouihan H.A., Bando J.K., Chawla A., Locksley R.M. (2011). Eosinophils sustain adipose alternatively activated macrophages associated with glucose homeostasis. Science.

[141] Qiu Y., Nguyen K.D., Odegaard J.I., Cui X., Tian X., Locksley R.M., Palmiter R.D., Chawla A. (2014). Eosinophils and type 2 cytokine signaling in macrophages orchestrate development of functional beige fat. Cell.

[142] Fabbiano S., Suárez-Zamorano N., Rigo D., Veyrat-Durebex C., Stevanovic Dokic A., Colin D.J., Trajkovski M. (2016). Caloric Restriction Leads to Browning of White Adipose Tissue through Type 2 Immune Signaling. Cell Metab..

[143] Zhang Y., Yang P., Cui R., Zhang M., Li H., Qian C., Sheng C., Qu S., Bu L. (2015). Eosinophils Reduce Chronic Inflammation in Adipose Tissue by Secreting Th2 Cytokines and Promoting M2 Macrophages Polarization. Int. J. Endocrinol..

[144] Huang Z., Zhong L., Lee J.T.H., Zhang J., Wu D., Geng L., Wang Y., Wong C.M., Xu A. (2017). The FGF21-CCL11 Axis Mediates Beiging of White Adipose Tissues by Coupling Sympathetic Nervous System to Type 2 Immunity. Cell Metab..

[145] Kim H.J., Alonzo E.S., Dorothee G., Pollard J.W., Sant’Angelo D.B. (2010). Selective depletion of eosinophils or neutrophils in mice impacts the efficiency of apoptotic cell clearance in the thymus. PLoS ONE.

[146] Wang H.-B., Ghiran I., Matthaei K., Weller P.F. (2007). Airway Eosinophils: Allergic Inflammation Recruited Professional Antigen-Presenting Cells. J. Immunol..

[147] Jacobsen E.A., Ochkur S.I., Pero R.S., Taranova A.G., Protheroe C.A., Colbert D.C., Lee N.A., Lee J.J. (2008). Allergic pulmonary inflammation in mice is dependent on eosinophil-induced recruitment of effector T cells. J. Exp. Med..

[148] Spencer L.A., Szela C.T., Perez S.A.C., Kirchhoffer C.L., Neves J.S., Radke A.L., Weller P.F. (2008). Human eosinophils constitutively express multiple Th1, Th2, and immunoregulatory cytokines that are secreted rapidly and differentially. J. Leukoc. Biol..

[149] Marc-Malovrh M., Camlek L., Škrgat S., Kern I., Fležar M., Dežman M., Korošec P. (2020). Elevated eosinophils, IL5 and IL8 in induced sputum in asthma patients with accelerated FEV1 decline. Respir. Med..

[150] Willebrand R., Voehringer D. (2016). IL-33-Induced Cytokine Secretion and Survival of Mouse Eosinophils Is Promoted by Autocrine GM-CSF. PLoS ONE.

[151] Barretto K.T., Swanson C.M., Nguyen C.L., Annis D.S., Esnault S.J., Mosher D.F., Johansson M.W. (2018). Control of cytokine-driven eosinophil migratory behavior by TGF-beta-induced protein (TGFBI) and periostin. PLoS ONE.

[152] Heredia J.E., Mukundan L., Chen F.M., Mueller A.A., Deo R.C., Locksley R.M., Rando T.A., Chawla A. (2013). Type 2 innate signals stimulate fibro/adipogenic progenitors to facilitate muscle regeneration. Cell.

[153] Goh Y.P.S., Henderson N.C., Heredia J.E., Eagle A.R., Odegaard J.I., Lehwald N., Nguyen K.D., Sheppard D., Mukundan L., Locksley R.M. (2013). Eosinophils secrete IL-4 to facilitate liver regeneration. Proc. Natl. Acad. Sci. USA.

[154] Reichman H., Moshkovits I., Itan M., Pasmanik-Chor M., Vogl T., Roth J., Munitz A. (2017). Transcriptome profiling of mouse colonic eosinophils reveals a key role for eosinophils in the induction of s100a8 and s100a9 in mucosal healing. Sci. Rep..

[155] Puxeddu I., Berkman N., Ribatti D., Bader R., Haitchi H.M., Davies D.E., Howarth P.H., Levi-Schaffer F. (2010). Osteopontin is expressed and functional in human eosinophils. Allergy Eur. J. Allergy Clin. Immunol..

[156] Nhu Q.M., Aceves S.S. (2017). Tissue Remodeling in Chronic Eosinophilic Esophageal Inflammation: Parallels in Asthma and Therapeutic Perspectives. Front. Med..

[157] Abdala-Valencia H., Coden M.E., Chiarella S.E., Jacobsen E.A., Bochner B.S., Lee J.J., Berdnikovs S. (2018). Shaping eosinophil identity in the tissue contexts of development, homeostasis, and disease. J. Leukoc. Biol..

[158] Fulkerson P.C., Rothenberg M.E. (2018). Eosinophil Development, Disease Involvement, and Therapeutic Suppression. Adv. Immunol..

[159] Marichal T., Mesnil C., Bureau F. (2017). Homeostatic Eosinophils: Characteristics and Functions. Front. Med..

[160] Rosenberg H.F., Dyer K.D., Foster P.S. (2013). Eosinophils: Changing perspectives in health and disease. Nat. Rev. Immunol..

[161] Chu D.K., Jimenez-Saiz R., Verschoor C.P., Walker T.D., Goncharova S., Llop-Guevara A., Shen P., Gordon M.E., Barra N.G., Bassett J.D. (2014). Indigenous enteric eosinophils control DCs to initiate a primary Th2 immune response in vivo. J. Exp. Med..

[162] Abdala Valencia H., Loffredo L.F., Misharin A.V., Berdnikovs S. (2016). Phenotypic plasticity and targeting of Siglec-FhighCD11clow eosinophils to the airway in a murine model of asthma. Allergy Eur. J. Allergy Clin. Immunol..

[163] Aoki A., Hirahara K., Kiuchi M., Nakayama T. (2021). Eosinophils: Cells known for over 140 years with broad and new functions. Allergol. Int..

[164] Mesnil C., Raulier S., Paulissen G., Xiao X., Birrell M.A., Pirottin D., Janss T., Starkl P., Ramery E., Henket M. (2016). Lung-resident eosinophils represent a distinct regulatory eosinophil subset. J. Clin. Investig..

[165] Ogawa M., Ishihara T., Isobe Y., Kato T., Kuba K., Imai Y., Uchino Y., Tsubota K., Arita M. (2020). Eosinophils promote corneal wound healing via the 12/15-lipoxygenase pathway. FASEB J..

[166] Brigger D., Riether C., van Brummelen R., Mosher K.I., Shiu A., Ding Z., Zbären N., Gasser P., Guntern P., Yousef H. (2020). Eosinophils regulate adipose tissue inflammation and sustain physical and immunological fitness in old age. Nat. Metab..

[167] Kuang F.L. (2020). Approach to Patients with Eosinophilia. Med. Clin. N. Am..

[168] Khoury P., Akuthota P., Ackerman S.J., Arron J.R., Bochner B.S., Collins M.H., Kahn J.E., Fulkerson P.C., Gleich G.J., Gopal-Srivastava R. (2018). Revisiting the NIH Taskforce on the Research needs of Eosinophil-Associated Diseases (RE-TREAD). J. Leukoc. Biol..

[169] Dellon E.S., Liacouras C.A., Molina-Infante J., Furuta G.T., Spergel J.M., Zevit N., Spechler S.J., Attwood S.E., Straumann A., Aceves S.S. (2018). Updated International Consensus Diagnostic Criteria for Eosinophilic Esophagitis: Proceedings of the AGREE Conference. Gastroenterology.

[170] Lwin T., Melton S.D., Genta R.M. (2011). Eosinophilic gastritis: Histopathological characterization and quantification of the normal gastric eosinophil content. Mod. Pathol..

[171] Dellon E.S., Hirano I. (2018). Epidemiology and Natural History of Eosinophilic Esophagitis. Gastroenterology.

[172] Lyles J., Rothenberg M. (2019). Role of genetics, environment, and their interactions in the pathogenesis of eosinophilic esophagitis. Curr. Opin. Immunol..

[173] Cañas J.A., Tabares A., Barbero C., García-Sánchez D., Sastre B., Rodrigo-Muñoz J.M., Mahíllo-Fernández I., Rayo A., Borrell B., Cilleruelo M.L. (2020). Proton-pump Inhibitor Response Prediction Using Esophageal microRNAs in Children With Eosinophilic Esophagitis. J. Pediatric Gastroenterol. Nutr..

[174] Manohar M., Kandikattu H.K., Upparahalli Venkateshaiah S., Yadavalli C.S., Mishra A. (2021). Eosinophils in the pathogenesis of pancreatic disorders. Semin. Immunopathol..

[175] Manohar M., Verma A.K., Venkateshaiah S.U., Sanders N.L., Mishra A. (2017). Pathogenic mechanisms of pancreatitis. World J. Gastrointest. Pharmacol. Ther..

[176] Kakodkar S., Omar H., Cabrera J., Chi K. (2015). Eosinophilic Pancreatitis Diagnosed With Endoscopic Ultrasound. ACG Case Rep. J..

[177] Bastid C., Sahel J., Choux R., Payan M.J., Sarles H. (1990). Eosinophilic pancreatitis: Report of a case. Pancreas.

[178] Grisaru-Tal S., Itan M., Klion A.D., Munitz A. (2020). A new dawn for eosinophils in the tumour microenvironment. Nat. Rev. Cancer.

[179] Reichman H., Itan M., Rozenberg P., Yarmolovski T., Brazowski E., Varol C., Gluck N., Shapira S., Arber N., Qimron U. (2019). Activated eosinophils exert antitumorigenic activities in colorectal cancer. Cancer Immunol. Res..

[180] Carretero R., Sektioglu I.M., Garbi N., Salgado O.C., Beckhove P., Hämmerling G.J. (2015). Eosinophils orchestrate cancer rejection by normalizing tumor vessels and enhancing infiltration of CD8 + T cells. Nat. Immunol..

[181] Hollande C., Boussier J., Ziai J., Nozawa T., Bondet V., Phung W., Lu B., Duffy D., Paradis V., Mallet V. (2019). Inhibition of the dipeptidyl peptidase DPP4 (CD26) reveals IL-33-dependent eosinophil-mediated control of tumor growth. Nat. Immunol..

[182] D’Angelo G., Hotz A.M., Todeschin P. (2008). Acute lymphoblastic leukemia with hypereosinophilia and 9p21 deletion: Case report and review of the literature. Lab. Hematol..

[183] Sahu K.K., Malhotra P., Khadwal A., Sachdeva M.S., Sharma P., Varma N., Varma S.C. (2015). Hypereosinophilia in Acute Lymphoblastic Leukemia: Two Cases with Review of Literature. Indian J. Hematol. Blood Transfus..

[184] Diny N.L., Rose N.R., Čiháková D. (2017). Eosinophils in Autoimmune Diseases. Front. Immunol..

[185] Hallam C., Pritchard D.I., Trigg S., Eady R.P. (1982). Rat eosinophil-mediated antibody-dependent cellular cytotoxicity: Investigations of the mechanisms of target cell lysis and inhibition by glucocorticoids. Clin. Exp. Immunol..

[186] Noguchi H., Kephart G.M., Colby T.V., Gleich G.J. (1992). Tissue eosinophilia and eosinophil degranulation in syndromes associated with fibrosis. Am. J. Pathol..

[187] del Pozo V., de Andrés B., Martín E., Cárdaba B., Fernández J.C., Gallardo S., Tramón P., Palomino P., Lahoz C., Leyva-Cobian F. (1992). Eosinophil as antigen-presenting cell: Activation of T cell clones and T cell hybridoma by eosinophils after antigen processing. Eur. J. Immunol..

[188] Bernard P., Venot J., Constant F., Bonnetblane J.M. (1987). Blood eosinophilia as a severity marker for bullous pemphigoid. J. Am. Acad. Dermatol..

[189] Tsukadaira A. (1999). Eosinophil active cytokines and surface analysis of eosinophils in churg-strauss syndrome. Allergy Asthma Proc..

[190] Loktionov A. (2019). Eosinophils in the gastrointestinal tract and their role in the pathogenesis of major colorectal disorders. World J. Gastroenterol..

[191] Carey E.J., Ali A.H., Lindor K.D. (2015). Primary biliary cirrhosis. Lancet.

[192] Kang E.G., Narayana P.K., Pouliquen I.J., Lopez M.C., Ferreira-Cornwell M.C., Getsy J.A. (2020). Efficacy and safety of mepolizumab administered subcutaneously for moderate to severe atopic dermatitis. Allergy.

[193] Fokkens W.J., Lund V.J., Hopkins C., Hellings P.W., Kern R., Reitsma S., Toppila-Salmi S., Bernal-Sprekelsen M., Mullol J., Alobid I. (2020). European Position Paper on Rhinosinusitis and Nasal Polyps 2020. Rhinology.

[194] Simon H.U., Yousefi S., Schranz C., Schapowal A., Bachert C., Blaser K. (1997). Direct demonstration of delayed eosinophil apoptosis as a mechanism causing tissue eosinophilia. J. Immunol..

[195] Bochner B.S., Stevens W.W. (2021). Biology and function of eosinophils in chronic rhinosinusitis with or without nasal polyps. Allergy Asthma Immunol. Res..

[196] Tay T.R., Radhakrishna N., Hore-Lacy F., Smith C., Hoy R., Dabscheck E., Hew M. (2016). Comorbidities in difficult asthma are independent risk factors for frequent exacerbations, poor control and diminished quality of life. Respirology.

[197] Denlinger L.C., Phillips B.R., Ramratnam S., Ross K., Bhakta N.R., Cardet J.C., Castro M., Peters S.P., Phipatanakul W., Aujla S. (2017). Inflammatory and comorbid features of patients with severe asthma and frequent exacerbations. Am. J. Respir. Crit. Care Med..

[198] Ponikau J.U., Sherris D.A., Kephart G.M., Kern E.B., Gaffey T.A., Tarara J.E., Kita H. (2003). Features of airway remodeling and eosinophilic inflammation in chronic rhinosinusitis: Is the histopathology similar to asthma?. J. Allergy Clin. Immunol..

[199] Fahy J.V. (2015). Type 2 inflammation in asthma-present in most, absent in many. Nat. Rev. Immunol..

[200] Wardlaw A.J., Dunnette S., Gleich G.J., Collins J.V., Kay A.B. (1988). Eosinophils and mast cells in bronchoalveolar lavage in subjects with mild asthma. Relationship to bronchial hyperreactivity. Am. Rev. Respir. Dis..

[201] Wardlaw A.L. (1999). Molecular basis for selective eosinophil trafficking in asthma: A multistep paradigm. J. Allergy Clin. Immunol..

[202] Nakagome K., Nagata M. (2018). Involvement and Possible Role of Eosinophils in Asthma Exacerbation. Front. Immunol..

[203] Walsh G.M. (2013). Eosinophil apoptosis and clearance in asthma. J. Cell Death.

[204] Anwar A.R.F., Moqbel R., Walsh G.M., Kay A.B., Wardlaw A.J. (1993). Adhesion to fibronectin prolongs eosinophil survival. J. Exp. Med..

[205] Green R.H., Brightling C.E., McKenna S., Hargadon B., Parker D., Bradding P., Wardlaw A.J., Pavord I.D. (2002). Asthma exacerbations and sputum eosinophil counts: A randomised controlled trial. Lancet.

[206] Demarche S.F., Schleich F.N., Paulus V.A., Henket M.A., Van Hees T.J., Louis R.E. (2017). Asthma Control and Sputum Eosinophils: A Longitudinal Study in Daily Practice. J. Allergy Clin. Immunol. Pract..

[207] Hanania N.A., Alpan O., Hamilos D.L., Condemi J.J., Reyes-Rivera I., Zhu J., Rosen K.E., Eisner M.D., Wong D.A., Busse W. (2011). Omalizumab in severe allergic asthma inadequately controlled with standard therapy: A randomized trial. Ann. Intern. Med..

[208] Szefler S.J., Wenzel S., Brown R., Erzurum S.C., Fahy J.V., Hamilton R.G., Hunt J.F., Kita H., Liu A.H., Panettieri R.A. (2012). Asthma outcomes: Biomarkers. J. Allergy Clin. Immunol..

[209] Ortega H., Katz L., Gunsoy N., Keene O., Yancey S. (2015). Blood eosinophil counts predict treatment response in patients with severe eosinophilic asthma. J. Allergy Clin. Immunol..

[210] Zeiger R.S., Schatz M., Dalal A.A., Chen W., Sadikova E., Suruki R.Y., Kawatkar A.A., Qian L. (2017). Blood Eosinophil Count and Outcomes in Severe Uncontrolled Asthma: A Prospective Study. J. Allergy Clin. Immunol. Pract..

[211] Inoue Y., Kaner R.J., Guiot J., Maher T.M., Tomassetti S., Moiseev S., Kuwana M., Brown K.K. (2020). Diagnostic and Prognostic Biomarkers for Chronic Fibrosing Interstitial Lung Diseases With a Progressive Phenotype. Chest.

[212] Travis W.D., Costabel U., Hansell D.M., King T.E., Lynch D.A., Nicholson A.G., Ryerson C.J., Ryu J.H., Selman M., Wells A.U. (2013). An official American Thoracic Society/European Respiratory Society statement: Update of the international multidisciplinary classification of the idiopathic interstitial pneumonias. Am. J. Respir. Crit. Care Med..

[213] Akuthota P., Weller P.F. (2012). Eosinophilic pneumonias. Clin. Microbiol. Rev..

[214] Guiot J., Moermans C., Henket M., Corhay J.L., Louis R. (2017). Blood Biomarkers in Idiopathic Pulmonary Fibrosis. Lung.

[215] Kuang F.L., Klion A.D. (2017). Biologic Agents for the Treatment of Hypereosinophilic Syndromes. J. Allergy Clin. Immunol. Pract..

[216] Fulkerson P.C., Rothenberg M.E. (2013). Targeting eosinophils in allergy, inflammation and beyond. Nat. Rev. Drug Discov..

[217] Ilmarinen P., Kankaanranta H. (2014). Eosinophil apoptosis as a therapeutic target in allergic asthma. Basic Clin. Pharmacol. Toxicol..

[218] Gotlib J. (2017). World Health Organization-defined eosinophilic disorders: 2017 update on diagnosis, risk stratification, and management. Am. J. Hematol..

[219] Kankaanranta H., Zhang X., Tumelius R., Ruotsalainen M., Haikala H., Nissinen E., Moilanen E. (2007). Antieosinophilic activity of simendans. J. Pharmacol. Exp. Ther..

[220] Izumi K., Bieber K., Ludwig R.J. (2019). Current clinical trials in pemphigus and pemphigoid. Front. Immunol..

[221] Fala L. (2016). Nucala (Mepolizumab): First IL-5 Antagonist Monoclonal Antibody FDA Approved for Maintenance Treatment of Patients with Severe Asthma. Am. Health Drug Benefits.

[222] Zhang J., Kuvelkar R., Murgolo N.J., Taremi S.S., Chou C.C., Wang P., Billah M.M., Egan R.W. (1999). Mapping and characterization of the epitope(s) of Sch 55700, a humanized mAb, that inhibits human IL-5. Int. Immunol..

[223] Castro M., Zangrilli J., Wechsler M.E., Bateman E.D., Brusselle G.G., Bardin P., Murphy K., Maspero J.F., O’Brien C., Korn S. (2015). Reslizumab for inadequately controlled asthma with elevated blood eosinophil counts: Results from two multicentre, parallel, double-blind, randomised, placebo-controlled, phase 3 trials. Lancet Respir. Med..

[224] Flood-Page P.T., Menzies-Gow A.N., Kay A.B., Robinson D.S. (2003). Eosinophil’s role remains uncertain as anti-interleukin-5 only partially depletes numbers in asthmatic airway. Am. J. Respir. Crit. Care Med..

[225] Liu W., Ma X., Zhou W. (2019). Adverse events of benralizumab in moderate to severe eosinophilic asthma: A meta-analysis. Medicine.

[226] Kolbeck R., Kozhich A., Koike M., Peng L., Andersson C.K., Damschroder M.M., Reed J.L., Woods R., Dall’acqua W.W., Stephens G.L. (2010). MEDI-563, a humanized anti-IL-5 receptor alpha mAb with enhanced antibody-dependent cell-mediated cytotoxicity function. J. Allergy Clin. Immunol..

[227] Maggi L., Rossettini B., Montaini G., Matucci A., Vultaggio A., Mazzoni A., Palterer B., Parronchi P., Maggi E., Liotta F. (2018). Omalizumab dampens type 2 inflammation in a group of long-term treated asthma patients and detaches IgE from FcεRI. Eur. J. Immunol..

[228] Ridolo E., Pucciarini F., Nizi M.C., Makri E., Kihlgren P., Panella L., Incorvaia C. (2020). Mabs for treating asthma: Omalizumab, mepolizumab, reslizumab, benralizumab, dupilumab. Hum. Vaccines Immunother..

[229] Delgado J., Dávila I.J., Domínguez-Ortega J. (2021). Clinical recommendations for the management of biological treatments in severe asthma patients: A consensus statement. J. Investig. Allergol. Clin. Immunol..

[230] Dorey-Stein Z.L., Shenoy K.V. (2021). Tezepelumab as an emerging therapeutic option for the treatment of severe asthma: Evidence to date. Drug Des. Dev. Ther..

[231] 2021 GINA Main Report-Global Initiative for Asthma-GINA. https://ginasthma.org/gina-reports/.

[232] Siddiqui S.H., Guasconi A., Vestbo J., Jones P., Agusti A., Paggiaro P., Wedzicha J.A., Singh D. (2015). Blood eosinophils: A biomarker of response to extrafine beclomethasone/formoterol in chronic obstructive pulmonary disease. Am. J. Respir. Crit. Care Med..

[233] Park H.S., Lee S.H., Lee S.Y., Kim M.K., Lee B.J., Werkström V., Barker P., Zangrilli J.G. (2019). Efficacy and Safety of Benralizumab for Korean Patients With Severe, Uncontrolled Eosinophilic Asthma. Allergy Asthma Immunol. Res..

[234] McGregor M.C., Krings J.G., Nair P., Castro M. (2019). Role of biologics in asthma. Am. J. Respir. Crit. Care Med..

[235] Jackson D.J., Korn S., Mathur S.K., Barker P., Meka V.G., Martin U.J., Zangrilli J.G. (2020). Safety of Eosinophil-Depleting Therapy for Severe, Eosinophilic Asthma: Focus on Benralizumab. Drug Saf..

[236] Gleich G.J., Klion A.D., Lee J.J., Weller P.F. (2013). The consequences of not having eosinophils. Allergy.

